# A donor–acceptor zwitterion as a self-assembled hole-selective layer for highly efficient tin-based perovskite solar cells

**DOI:** 10.1039/d5sc08439c

**Published:** 2025-12-29

**Authors:** Qianqian Chang, Guosen Zhang, Diwei Zhang, Peng Lin, Jingjing Li, Xurang Wang, Tianci Gu, Jingying Lin, Yuan Lin, Xiaozhen Li, Mingwei An, Yu Cao, Chengbo Tian, Yang Wang

**Affiliations:** a Strait Institute of Flexible Electronics (SIFE, Future Technologies), Fujian Key Laboratory of Flexible Electronics, College of Physics and Energy, Fujian Normal University and Strait Laboratory of Flexible Electronics (SLoFE) Fuzhou 350117 Fujian P. R. China ifedwzhang@fjnu.edu.cn ifemwan@fjnu.edu.cn ifeyucao@fjnu.edu.cn ifewangy@fjnu.edu.cn; b Xiamen Key Laboratory of Optoelectronic Materials and Advanced Manufacturing, Institute of Luminescent Materials and Information Displays, College of Materials Science and Engineering, Huaqiao University Xiamen 361021 China cbtian@hqu.edu.cn

## Abstract

The development of tin-based perovskite solar cells (TPSCs) has lagged far behind that of their lead-based counterparts. Although high-efficiency TPSCs have been reported in recent years, they are all based on poly(3,4-ethylenedioxythiophene):poly(styrenesulfonate) (PEDOT:PSS) as the hole-selective layer (HSL), whose strong acidity and hygroscopicity are undoubtedly highly detrimental to the long-term stability of the devices. Here, a donor–acceptor-type zwitterionic molecule (PyPs) was designed by employing a triphenylamine donor and a benzo[*c*][1,2,5]thiadiazole acceptor as the molecular backbone, functionalized with a pyridinium sulfonate terminal group. The ionic sulfonate group in PyPs not only exhibits stronger coordination with indium tin oxide (ITO), enabling uniform surface coverage and improved energy-level alignment, but also assists the growth and defect passivation of a tin perovskite. As a result, high-quality Sn-based perovskite films can be obtained along with accelerated interfacial charge extraction and suppressed non-radiative recombination losses. Encouragingly, PyPs-based devices deliver a champion power conversion efficiency (PCE) of 12.18%, representing the highest efficiency reported to date for TPSCs based on alternative HSLs to PEDOT:PSS. Moreover, unencapsulated PyPs-based devices retain 90% of their initial PCE after 1800 h of storage. This work highlights the potential of rational molecular design in the exploration of alternative HSLs for efficient and stable TPSCs.

## Abstract 2

A donor–acceptor zwitterion, PyPs, has been proposed to regulate the crystallization kinetics and hole-selective contact in tin-based perovskite solar cells (TPSCs), achieving a record-breaking efficiency of 12.18%.

## Graphical abstract

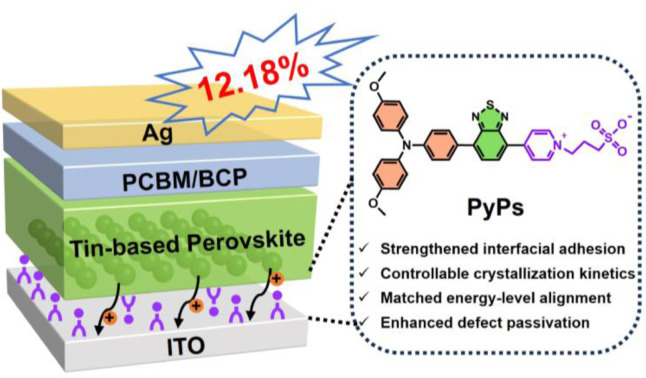

## Introduction

Metal halide perovskites have attracted enormous interest from both academics and industry for next-generation photovoltaic technologies, thanks to their solution-processability, low cost and defect tolerance.^1–4^ However, the toxicity of lead ions causes environmental hazards and health concerns, which impede their commercialization.^5,6^ To mitigate this issue, tin-based perovskites have emerged as compelling lead-free alternatives, exhibiting advantageous optoelectronic properties: optimal Shockley–Queisser bandgaps, high charge-carrier mobilities and low exciton binding energies.^7–9^ Nevertheless, tin (Sn)-based perovskite solar cells (TPSCs) underperform compared to their lead counterparts, mainly because of the uncontrollable crystallization process and unstable interfacial properties, which include the facile oxidation of Sn^2+^ and energy-level mismatch between charge selective layers (CSLs) and the perovskite layer.^10–13^ Alongside parallel research on perovskites and electron selective layers (ESLs),^14–17^ hole-selective layer (HSL) research is relatively scarce in TPSCs and is anticipated to bring further remarkable progress in the future.

To date, state-of-the-art, high-performance TPSCs with PCEs exceeding 15% have utilized a p–i–n architecture, and poly(3,4-ethylene dioxythiophene) polystyrene sulfonate (PEDOT:PSS) serves as the standard HSL in current devices.^4,18–20^ However, the interfacial mismatch between a Sn-based perovskite and PEDOT:PSS could induce inferior interfacial contact, resulting in considerable open-circuit voltage (*V*_OC_) losses.^21–23^ Worse still, its inherent electrochemical properties, such as acidity, hygroscopicity and anisotropic charge transport characteristics, severely limit the performance of PEDOT-based devices and long-term stability.^24–27^ Therefore, there is an urgent need for the rational design and development of novel HSLs that can effectively address these fundamental limitations and unlock the full potential of Sn-based perovskite photovoltaics. Numerous hole transport materials (HTMs) have been widely employed in efficient TPSCs, including NiO_*x*_, PTAA, and various novel self-assembled molecules (SAMs).^28–33^ The Diau group first reported PTAA-based TPSCs, where modifying the surface hydrophobicity of PTAA and passivating the crystal interface *via* π–π interactions with PEAI led to high-quality perovskite films and enhanced device efficiency and stability.^30^ The same team also utilized SAMs such as 2PACz and MeO-2PACz to modify ITO as the HTL, achieving a champion PCE of 6.5% with MeO-2PACz.^34^ Abate *et al.* designed and synthesized a SAM named Th-2EPT, featuring a pyrimidine core and phosphonic acid anchoring group. This SAM improved the interface of tin-based perovskites and enabled one-step perovskite deposition, resulting in a PCE of 8.2%.^35^ Furthermore, several new SAMs with cyanoacrylic acid anchor groups, including TQxD and TPAT-CA, have been developed.^36,37^ Despite these advances, the reported device efficiencies have not yet exceeded 10%. The fundamental reasons for this lie in the fact that most HSLs struggle to simultaneously meet the following critical requirements: (1) good wettability towards the Sn-based perovskite precursor solution;^34^ (2) well-matched energy-level alignment with the Sn-based perovskite;^38^ (3) the capability to regulate the growth and crystallization of the Sn-based perovskite film while effectively passivating defects and suppressing the oxidation of Sn^2+^ ions at the buried interface.^38^

Zwitterions are a type of ionic molecule possessing both positive and negative electronic charges within the same molecule.^39^ The oppositely charged ions, connected covalently to each other, produce stable dipoles in the same molecule.^40^ Meanwhile, such molecules bearing ‘inner salts’ are superior hydrophilic materials, which have been extensively applied as interfacial materials in optoelectronic devices.^41–43^ Similarly, in the context of PSCs, zwitterions have been widely reported as additives,^44,45^ passivators,^46,47^ or interface modification materials,^48,49^ sufficiently demonstrating the application potential of this class of molecules in regulating the performance of PSCs. However, studies on zwitterions as single-component CSLs remain scarce to date, especially for TPSCs. Exhibiting unique properties, we believe that zwitterions may offer a promising avenue for constructing efficient HSLs through rational molecular design.

Therefore, in this work, a donor–acceptor zwitterion was designed and synthesized by incorporating triphenylamine as the donor unit, benzo[*c*][1,2,5]thiadiazole as the acceptor unit, and a pyridinium sulfonate group as the terminal group (PyPs). For comparison, a non-ionic molecule with pyridine as the end group was also synthesized (Py). Compared with Py, the zwitterion PyPs features a stronger dipole moment and superior wettability toward a Sn-based perovskite precursor solution. Moreover, the sulfonate group in PyPs shows stronger anchoring ability toward ITO, realizing better coverage, which is conducive to the growth of high-quality Sn-based perovskite films with suppressed oxidation of Sn^2+^ to Sn^4+^. Additionally, the introduction of PyPs enables better energy-level alignment at the ITO/perovskite interface, accelerates interfacial carrier extraction and transfer, and minimizes non-radiative recombination losses. As a result, when PyPs served as an HSL in TPSCs, the PyPs-based device reached a champion efficiency of 12.18%, which is much higher than those of Py-based (10.22%) and pure ITO-based (8.60%) devices. Furthermore, the unencapsulated PyPs-based devices displayed excellent long-term stability, maintaining 90% of the initial efficiency after storage under N_2_ conditions for about 1800 h. This work provides a promising candidate for HSLs in a Sn-based perovskite and demonstrates that rational molecular design can unlock new levels of performance in TPSCs.

## Results and discussion

**Fig. 1:**
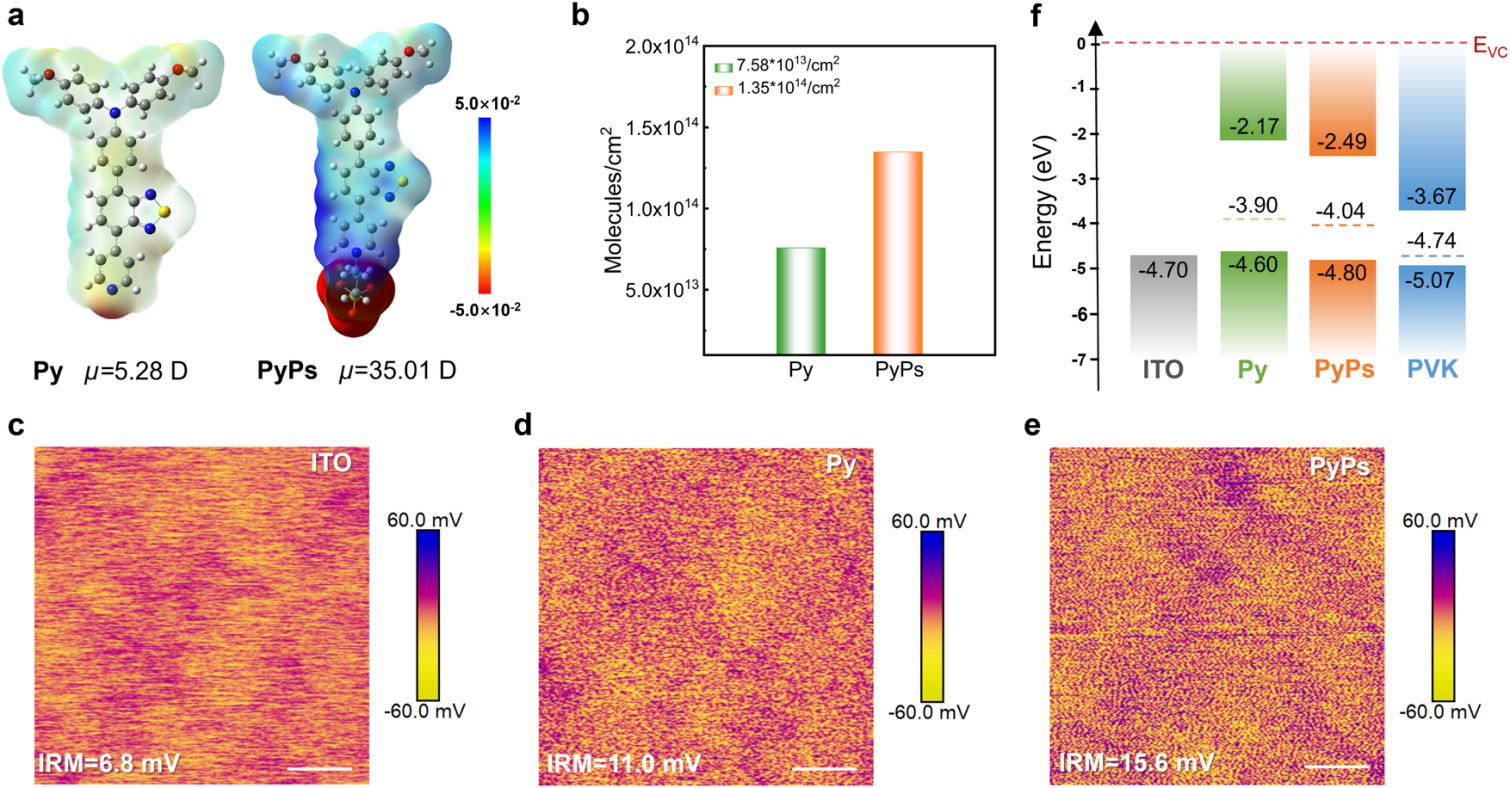
Molecular structure and interfacial properties. (a) The electrostatic potential (ESP) of Py and PyPs. (b) Bar chart illustrating the molecular surface coverage density of different molecules deposited on ITO/PyPs (Py) substrates. (c–e) KPFM images of ITO without and with Py and PyPs films (scale bar 500 nm). (f) Energy diagram comparing the alignment between the perovskite valence band edge and the HOMO energy levels of ITO/PyPs (Py) substrates from UPS measurements, referenced to the vacuum levels (*E*_vc_).

The zwitterion PyPs and Py were synthesized within three steps involving successive Suzuki coupling and ionization reaction, as shown in the SI (Scheme S1), which also shows their structural characterizations in Fig. S1–S9. Both Py and PyPs exhibit high thermal stability with decomposition temperatures above 230 °C (Fig. S10). Despite their broad intrinsic absorption (Fig. S11), both Py and PyPs induce negligible parasitic absorption when coated as ultrathin hole-selective layers on ITO substrates (Fig. S12). Density functional theory (DFT) was utilized to investigate the frontier molecular orbital (FMO) energy levels, dipole moments and surface electrostatic potentials (ESP) of the two molecules. Compared to Py, the additional sulfonate acid group (–SO_3_^−^) in PyPs slightly alters the HOMO and LUMO distribution, leading to lower corresponding energy levels (Fig. S13). Correspondingly, the cyclic voltammetry (CV) curves demonstrate identical HOMO level variation trends (Fig. S14). Meanwhile, as shown in Fig. 1a, –SO_3_^−^ groups tend to induce more negative charges, which may be due to the high electronegativity of sulfur and oxygen atoms.^50^ The molecular dipole moment of PyPs (35.01 D) is much larger than that of Py (5.28 D), which is advantageous for hole transfer at the ITO/perovskite interface.^51^

The coverage density and compactness of the two HSLs can be further compromised by the subsequent perovskite deposition if the anchoring strength is insufficient. We therefore estimated the robustness of the HSLs by using CV measurement as demonstrated in the literature.^52,53^ The areal density for each molecule on ITO increases from Py (7.57 × 10^13^ molecules cm^−2^) to PyPs (1.35 × 10^14^ molecules cm^−2^), indicating better coverage of PyPs on the ITO substrate (Fig. 1b and S15). This may be attributed to the improved adhesion toward the substrate for the ionic anchoring group in PyPs than the pyridine group in Py.^50^ Moreover, the elemental mapping distribution for energy dispersive X-ray spectroscopy (EDS) also demonstrates that more PyPs molecules can effectively anchor on the ITO substrate, forming a tighter film coverage (Fig. S16). Similarly, the atomic force microscopy (AFM) characterization shown in Fig. S17 and S18 indicates that the surface roughness of the ITO substrate is reduced, especially that treated with PyPs. Meanwhile, the water contact angle increases from the ITO to ITO/PyPs (Py) substrate (Fig. S19).

Subsequently, thin films of Py and PyPs were fabricated on ITO substrates by the spin-coating method to investigate a series of electrical properties, including conductivity and surface potential. The current–voltage (*J*–*V*) curves of hole-only devices (Fig. S20) indicate the enhanced conductivity of ITO after modification with the two molecules, particularly for PyPs. Simultaneously, Kelvin probe force microscopy (KPFM) was adopted to probe the vibration of the surface potential of ITO with and without PyPs or Py. As shown in Fig. 1c–e, the average surface potential of ITO increased from 6.8 mV to 15.6 mV after PyPs modification. This means that the PyPs-treated sample has a lowered work function (WF) and a weakened p-type feature, which is favorable for interfacial band alignment and hole transport.^54^ Ultraviolet photoelectron spectroscopy (UPS) and ultraviolet-visible (UV-vis) absorption spectroscopy were further employed to investigate the ITO/perovskite interface band alignment (Fig. S21 and S22) with the data shown in Fig. 1f and Table S1. The results show that the PyPs-treated ITO exhibits a larger work function and deeper HOMO energy level compared to the ITO and ITO/Py substrates. PyPs molecules anchor strongly to the ITO surface through coordination between the negatively charged sulfonic acid group (–SO_3_^−^) and the substrate. Furthermore, PyPs possesses a significant intrinsic dipole moment, which facilitates the formation of an oriented and stable dipole layer at the ITO/polymer interface. After the introduction of PyPs, the strong dipole moment of PyPs enhances charge rearrangement at the interface. In parallel, as confirmed by Kelvin probe force microscopy (KPFM), the oriented dipole layer shifts the local vacuum level, increasing the work function of the ITO substrate.^53^ This elevated work function optimizes the energy-level alignment between ITO and the tin-based perovskite layer, leading to a stronger built-in electric field at the charge–separation interface. Consequently, hole extraction is enhanced, contributing to a higher *V*_OC_ in the device.

**Fig. 2:**
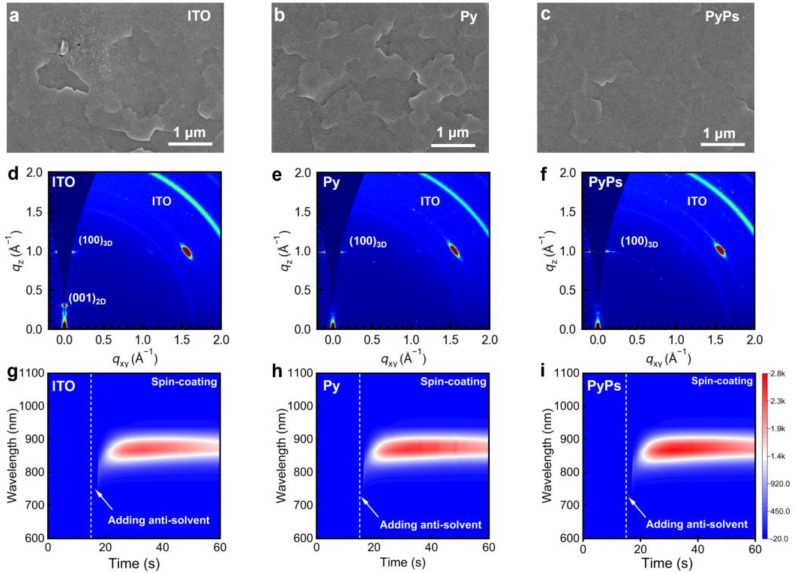
Effects of HSLs on perovskite crystallinity. (a–c) Top-view SEM images of perovskite films on ITO, Py and PyPs substrates, respectively. (d–f) GIWAXS patterns of the perovskite films on ITO, Py and PyPs substrates, respectively. (g–i) *In situ* PL spectra of perovskite films deposited on different substrates.

The wettability of the perovskite precursor solution on the substrate plays an important role in determining the perovskite film quality. PyPs shows a smaller contact angle than that of Py (8.4° *versus* 14.3°, Fig. S23), which can be attributed to the enhanced amphiphilicity of the zwitterion PyPs.^55^ Therefore, perovskite deposition is more convenient on these highly wettable substrates, and the interfacial contact will also be enhanced. The perovskite films deposited on different substrates exhibit similar absorption profiles and intensities (Fig. S22), suggesting a negligible difference in optical bandgap. However, the scanning electron microscopy (SEM) images (Fig. 2a–c) show that a smoother and more homogeneous surface of perovskites deposited on ITO/PyPs could be obtained. This can also be directly observed from the AFM images in Fig. S24, which display lower root mean square (RMS) values, especially for the perovskite based on ITO/PyPs. The crystallinity of perovskite films was further evaluated by grazing-incidence wide-angle X-ray scattering (GIWAXS) (Fig. 2d–f). The PyPs-based perovskite film exhibits brighter diffraction rings of the (100) plane than the Py- and ITO-based films, indicating enhanced perovskite crystallinity in the former, which is consistent with the X-ray diffraction (XRD) results (Fig. S25). In addition, the diffraction ring in GIWAXS at *q* values of 0.3 Å^−1^ corresponds to the (001) plane of the 2D perovskite.^56^ The significant suppression of the 2D peak in perovskite films based on PyPs (Py) (Fig. S26) indicates that both molecules promote a highly preferential crystal orientation, thereby enhancing film quality. To elucidate the crystallization mechanism of the perovskite on different substrates, *in situ* photoluminescence (PL) measurements were conducted during the spin-coating and annealing process (Fig. 2g–i and S28). Upon antisolvent dripping, the PL intensity of the perovskite deposited on PyPs increases rapidly and reaches the highest value among all substrates at the end of spin-coating (Fig. S27). This indicates that the –SO_3_^−^ group in PyPs promotes a higher nucleation density and stronger early-stage coordination with perovskite precursors, leading to more efficient initial radiative recombination. During the subsequent thermal annealing, the perovskite film on PyPs maintains a consistently higher PL intensity compared to those on Py or ITO (Fig. S28), suggesting improved crystallinity and effective defect passivation. These results demonstrate that the sulfonate group not only accelerates nucleation but also stabilizes the intermediate phases, thereby facilitating the formation of high-quality perovskite films with enhanced defect passivation. Cross-sectional SEM images reveal that denser and pinhole-free perovskite film grains can be formed on PyPs or Py compared to those on the ITO substrate (Fig. S29). Moreover, the KPFM of the perovskite based on PyPs features a much lower surface potential than those on other substrates, suggesting the mitigation of the p-type characteristics of the Sn-based perovskite film on PyPs (Fig. S30). Meanwhile, the PyPs-based film possesses a lower Urbach energy (*E*_U_) of 93.42 meV compared with that of the other films (Fig. S31), suggesting reduced energetic disorder in the perovskite film.^14^ Therefore, the high quality of the perovskite film can potentially reduce the bulk defect density and minimize interface non-radiative recombination losses.

**Fig. 3:**
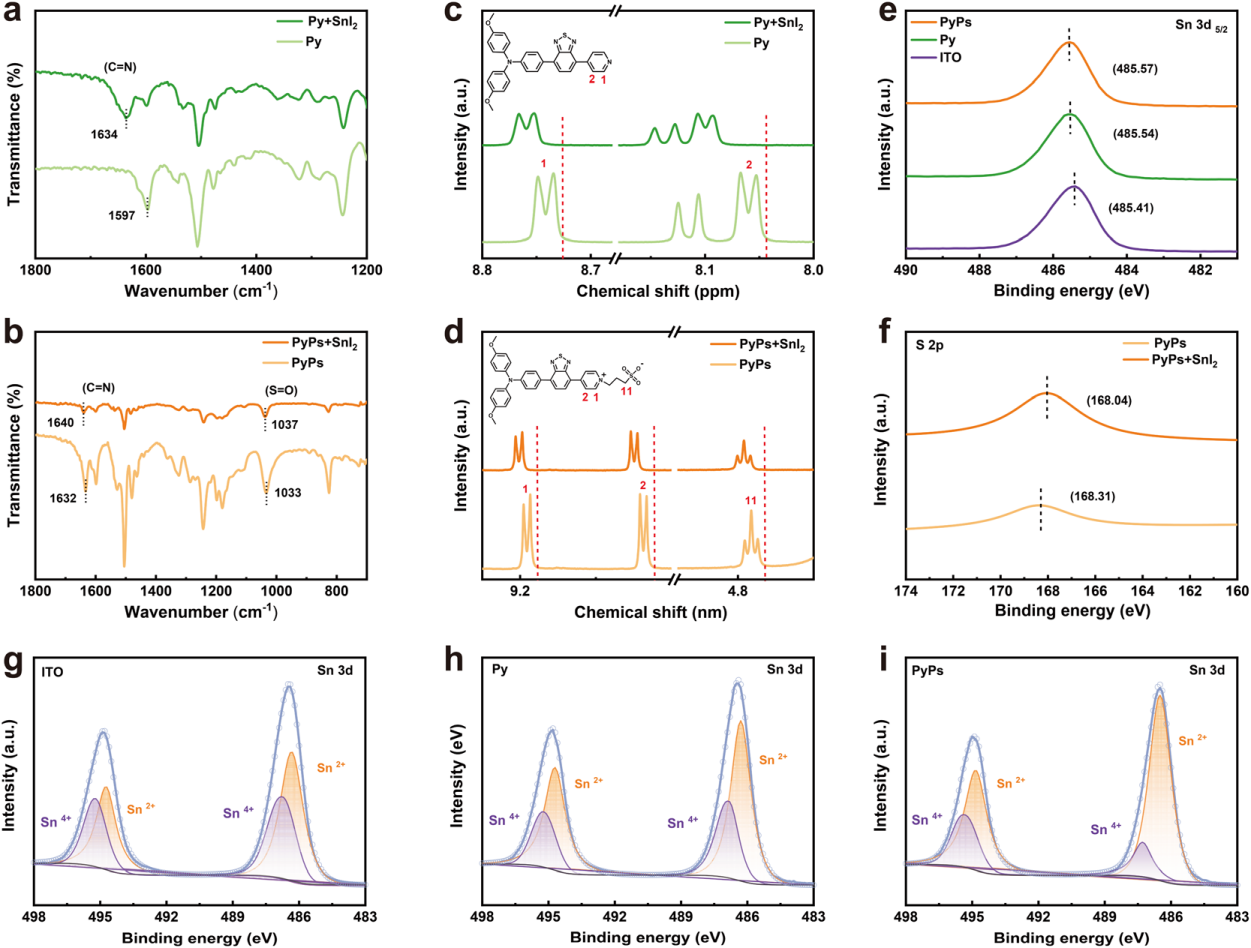
Interactions between the two molecules and the perovskite. FTIR spectra of (a) Py and (b) PyPs with or without mixing with SnI_2_. 1H NMR spectra of (c) Py and (d) PyPs with or without mixing with SnI_2_. (e) Sn 3d and (f) S 2p core level XPS spectra of the Sn-based perovskite with and without PyPs (Py) treatment. (g–i) Sn 3d core level XPS spectra were fitted to the contributions of Sn^2+^ and Sn^4+^ of ITO, Py and PyPs-based perovskite films, respectively.

To obtain insight into the passivation effect, which is highly related to the interactions between the two molecules and the perovskite, we utilized Fourier transform infrared spectroscopy (FTIR), ^1^H NMR and X-ray photoelectron spectroscopy (XPS) measurements to study the relative strength of the interactions. In the FTIR measurement (Fig. 3a and b), a small shift of *ν*_C=N_ from 1597 cm^−1^ to 1634 cm^−1^ can be found for Py mixing with SnI_2_, indicating the stronger interaction between the N atom in the pyridine group and the Sn^2+^ ion. In contrast, a remarkable discrepancy is observed for PyPs before and after blending with SnI_2_. Beyond the shift of the *ν*_C=N_ band from 1632 cm^−1^ to 1640 cm^−1^, the S=O of the characteristic –SO_3_^−^ group of PyPs also shifted at 1033 cm^−1^, further indicating the multiple interactions between PyPs and Sn^2+^ ions. In the 1H NMR test (Fig. 3c and d), adding SnI_2_ causes a clear downfield shift of the pyridine protons in Py. For PyPs, the shifts involve both pyridine protons and protons adjacent to the –SO_3_^−^ group, indicating synergistic interactions among its multiple functional groups. XPS measurements were conducted to check the interaction between the two molecules and the perovskites. Compared to the pristine perovskite film, both Py and PyPs show a more visible shift of the Sn 3d peaks to higher binding energy (Fig. 3e), confirming the stronger interactions between the PyPs and perovskite.^57^ Upon contact with the perovskite, the S 2p peak shifted to a higher binding energy (Fig. 3f), further supporting the interactions between the –SO_3_^−^ group and Sn^2+^ ion. Importantly, as depicted in Fig. 3g–i, the introduction of PyPs or Py greatly reduced the content of Sn^4+^. Notably, the Sn^4+^/Sn^2+^ proportion in the PyPs-based perovskite film is significantly reduced, compared with the pristine and Py-based perovskite films (Table S2), indicating that PyPs as the HSL can effectively inhibit the oxidation of Sn^2+^ in the Sn-based perovskite film. Based on these results, it can be concluded that the defects at the buried interface and bulk perovskite can be more effectively passivated by PyPs, which also reduces Sn^2+^ oxidation, substantially improving the optoelectronic quality of Sn-based perovskite thin films.

**Fig. 4:**
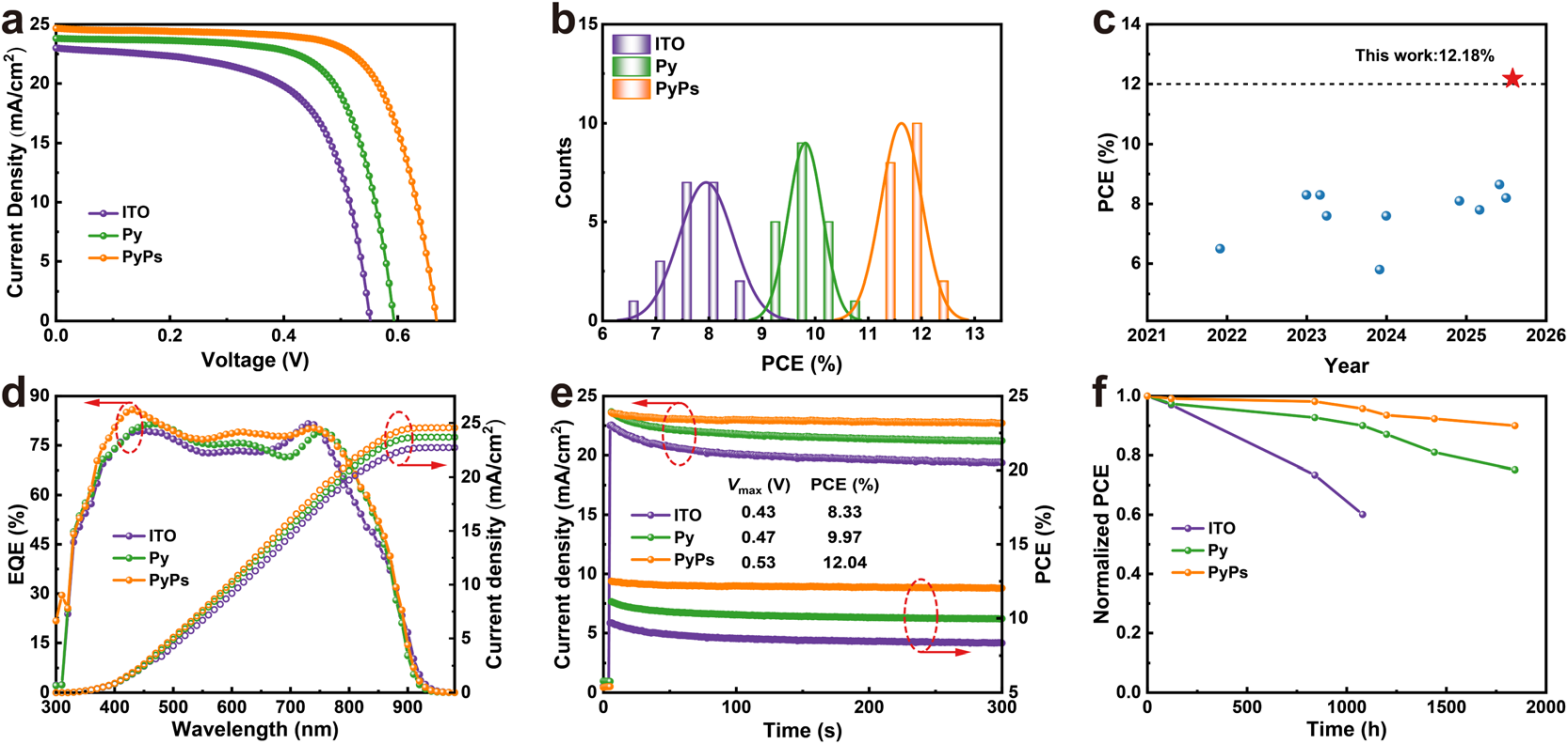
Photovoltaic performance and stability of PyPs (Py)-based TPSCs. (a) *J*–*V* curves of the best-performing TPSCs. (b) PCE distribution from 20 individual devices with different HSLs. (c) An efficiency comparison of TPSCs based on alternative HSLs to PEDOT:PSS reported in the literature with that achieved in our study (red star). (d) EQE spectra and the corresponding integrated photocurrents for the different devices. (e) Stabilized power output of the best devices under a voltage bias at the maximum power point. (f) Long-term stability tracking of the unencapsulated TPSCs with the control and PyPs (Py) under N_2_ conditions.

Based on these encouraging findings, Py and PyPs were used as HSLs for fabricating inverted TPSCs with a device structure of ITO/HSL/perovskite/PCBM/BCP/Ag, and the thickness of the perovskite film was about 300 nm (Fig. S32). The device fabrication details can be found in the SI. The *J*–*V* curves of the best-performing devices with the two molecules, measured under one sun illumination (AM1.5 sunlight, 100 mW cm^−2^), are displayed in Fig. 4a, and the average photovoltaic parameters are listed in Table 1. The ITO-based device exhibits a moderate PCE of 8.60%, with an open-circuit voltage (*V*_OC_) of 0.558 V, a short-circuit current (*J*_SC_) of 23.08 mA cm^−2^ and a fill factor (FF) of 66.79%. Upon using Py, the PCE is enhanced to 10.22%, based on values of *V*_OC_, *J*_SC_ and FF of 0.602 V, 23.95 mA cm^−2^ and 70.90%, respectively. In striking contrast, the PyPs-based device achieves a significantly higher PCE of 12.18%, with corresponding parameters of *V*_OC_, *J*_SC_ and FF all improving to 24.80 mA cm^−2^, 0.678 V and 72.34%, respectively. The significant fabrication reproducibility of PyPs (Py)-based TPSCs was confirmed through statistical analysis of photovoltaic parameters based on 20 independent devices (Fig. 4b, S33 and Table S3–S5). The optimized PyPs-based devices exhibit a narrow PCE distribution, suggesting improved reproducibility, which is especially important for further larger-scale manufacturing. The *J*–*V* curves of PyPs-based TPSCs with the best performance have almost the same photovoltaic parameters and exhibit negligible hysteresis (Fig. S34). After careful literature search and comparison, we found that this is the highest PCE reported to date for TPSCs based on alternative HSLs to PEDOT:PSS (Fig. 4c and Table S6). In addition, the external quantum efficiency (EQE) spectra of the corresponding devices are shown in Fig. 4d. The integrated photocurrents are calculated to be 22.71, 23.68 and 24.56 mA cm^−2^ for the devices based on ITO, Py and PyPs, respectively, which is in good agreement with the value of *J*_SC_ obtained from the *J*–*V* measurement. Fig. 4e shows the stable power output (tracking current densities under a bias voltage at the maximum power point) of PyPs-based devices. A stabilized PCE of 12.04% and a *J*_SC_ of 22.72 mA cm^−2^ were obtained, confirming the high reliability of the *J*–*V* scanning results.

**Table 1:** The best photovoltaic parameters of TPSCs based on different substrates

Device		*J*_SC_ (mA cm^−2^)	*V*_OC_ (V)	FF (%)	PCE (%)
ITO	Average	21.93 ± 0.63	0.559 ± 0.016	64.8 ± 3.1	7.95 ± 0.51
	Champion	23.08	0.558	66.79	8.60
Py	Average	23.97 ± 0.34	0.610 ± 0.017	67.2 ± 2.3	9.82 ± 0.32
	Champion	23.95	0.602	70.90	10.22
PyPs	Average	24.54 ± 0.25	0.668 ± 0.026	70.9 ± 1.4	11.62 ± 0.38
	Champion	24.80	0.678	72.34	12.18

Long-term stability is one of the most important performance indicators for future commercial applications of TPSCs. Hence, the device stability of the corresponding TPSCs (without and with PyPs or Py) was evaluated by tracking the PCE evolution under N_2_ conditions at room temperature. As shown in Fig. 4f, the PCE of the unencapsulated PyPs-based device exhibited enhanced stability, maintaining 90% of the initial efficiency after 1800 hours of aging. In contrast, the PCE values of the control and Py-based devices sharply decreased to below 65% (1000 h) and 75% of their initial PCEs, respectively. Regarding the long-term stability test, we also tracked the stability of the corresponding perovskite films based on different substrates. The XRD spectrum of perovskite films on PyPs is almost unchanged upon storage in N_2_ for 6 days, while the control and Py-based samples exhibited varying degrees of degradation (Fig. S35). Furthermore, long-term light stability tests under continuous laser illumination (ITO-side incidence) reveal that the PyPs-based film maintains stable PL intensity, whereas the other films show significant decay over time (Fig. S36). This suggests that the zwitterion PyPs layer effectively mitigates interfacial degradation pathways, thereby contributing to the overall device stability.

**Fig. 5:**
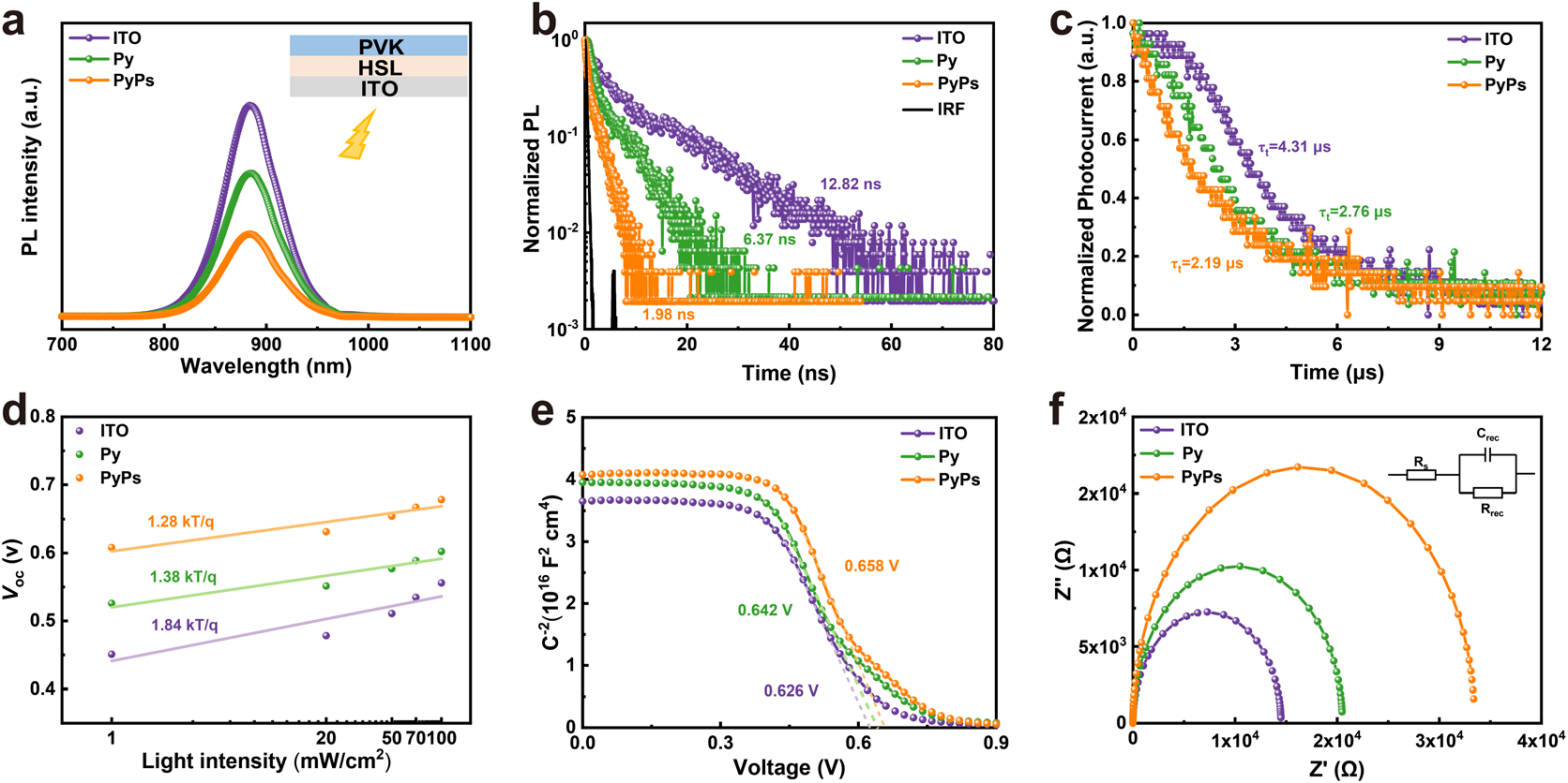
Carrier dynamics and recombination processes of different HSL-based films and devices. (a) PL and (b) TRPL spectra of perovskite deposited on ITO/PyPs (Py) upon excitation from the ITO side. (c) TPC decay curves and (d) the dependence of *V*_OC_ on light intensity for different substrate-based devices. (e) Mott–Schottky curves and (f) electrical impedance spectroscopy plots (inset: equivalent circuit model for the Nyquist plots) for different TPSCs.

To obtain insight into the interfacial charge dynamics and recombination process in the PyPs (Py)-based films and devices, steady-state photoluminescence (PL) and time-resolved PL (TRPL) measurements upon excitation from the ITO side were carried out. As illustrated in Fig. 5a, the PL quenching is more significant for the perovskite on the two molecules than on ITO, particularly for PyPs. Similarly, the TRPL analysis (Fig. 5b and Table S7) shows the decreasing average carrier lifetime from ITO (12.82 ns) to Py (6.37 ns) and PyPs (1.98 ns), suggesting reduced hole extraction between ITO and the perovskite. In contrast, top-side PL and TRPL measurements further reveal that the perovskite films on PyPs substrates exhibit enhanced PL intensity (Fig. S37) and a prolonged average carrier lifetime (Fig. S38 and Table S8). This indicates an improved film quality due to suppressed defect-assisted non-radiative recombination. Meanwhile, space charge limited current (SCLC) measurements of hole-only devices (ITO/HSL/perovskite/PTAA/Ag) were performed under dark conditions to determine the charge trap density of the perovskite. The PyPs-based device showed a trap-filled limit voltage (*V*_TFL_) of 0.283 V, much smaller than those of Py (0.327 V) and ITO (0.404 V) (Fig. S39). Therefore, the trap densities (*N*_t_) of the perovskite films deposited on ITO, Py and PyPs can be calculated to be 1.74 × 10^17^ cm^−3^, 1.41 × 10^17^ cm^−3^ and 1.22 × 10^17^ cm^−3^, respectively. The decreased trap density of the PyPs-based device proved clearly the effective passivation of the defects by the sulfonic group. Transient photocurrent (TPC) measurements were conducted to further investigate the charge-carrier dynamics in the whole devices. It was found that the charge-collection lifetimes of the ITO, Py and PyPs-based devices are 4.31 µs, 2.76 µs and 2.19 µs, respectively, indicating a facilitated charge-collection process in the PyPs-based device (Fig. 5c). The longer charge-recombination lifetime observed for PyPs in transient photovoltage (TPV) measurements (Fig. S40) further confirms this trend and verifies the effective mitigation of charge recombination at the ITO/perovskite interface. Furthermore, *V*_OC_ was measured by changing the intensity of incident light (*P*) from 1 to 100 mW cm^−2^. As shown in Fig. 5d, the PyPs-based device exhibits a smaller slope of 1.28 *kT*/*q* compared with those of Py (1.38 *kT*/*q*) and ITO (1.84 *kT*/*q*), indicating that the Shockley-Read-Hall (SRH) recombination is well suppressed for the PyPs-based device.^58,59^ Additionally, PyPs-based devices also exhibit lower leakage currents in the dark-state *J*–*V* curves (Fig. S41), indicating suppressed interface recombination and consistent with their superior *V*_OC_ and FF.

Subsequently, capacitance–voltage (*C*–*V*) and electrochemical impedance spectroscopy (EIS) measurements were employed to further investigate the charge transport and recombination dynamics in these devices. Fig. 5e presents the Mott–Schottky curves, elucidating the built-in potential (*V*_bi_) within the corresponding devices.^60^ By calculating the intercept of the *x*-axis, it is evident that the built-in potential of the PyPs-based device (0.658 V) surpasses that of the Py-based device (0.642 V) and the control device (0.626 V). The enhanced built-in potential has a stronger driving force to accelerate the separation and transport of charge carriers, which is a favorable indicator for achieving high *V*_OC_ in the device. Fig. 5f shows the fitted Nyquist plots of devices measured at *V*_OC_ under dark conditions, with the frequency range from 100 kHz to 1 Hz.^61^ All the devices showed low and similar series resistances (*R*_s_), which is beneficial for hole extraction.

However, gradually increased recombination resistance (*R*_rec_) values, from 1.45 × 10^4^ Ω to 2.05 × 10^4^ Ω and 3.34 × 10^4^ Ω, are observed for the devices based on ITO, Py and PyPs, respectively, indicating a decrease in the charge recombination rate. Therefore, these observations strongly indicate that PyPs can more effectively passivate surface defects, helping to significantly reduce non-radiative interface recombination processes.

## Conclusion

In summary, we designed and synthesized a donor–acceptor zwitterion, PyPs, as a HSL for TPSCs. Compared with the non-ionic molecule Py, the hydrophilicity of the perovskite precursor solution and the quality of the perovskite films deposited on PyPs were improved, mainly due to the dipole ionic group on the PyPs, thereby enhancing the contact and interaction with the perovskite. Meanwhile, the PyPs with improved energetic alignment could effectively passivate the buried interface defects, resulting in prolonged charge-carrier lifetime and suppressed non-radiative recombination. As a result, the devices based on PyPs achieved a record efficiency exceeding 12%, demonstrating the highest PCE reported to date for TPSCs based on alternative HSLs to PEDOT:PSS. Furthermore, PyPs contributed to the enhanced long-term stability of Sn-based perovskite films and devices, maintaining 90% of their initial PCE after 1800 hours of aging under N_2_ conditions. This work points to novel research directions for designing more advanced HSLs for high-performance TPSCs with excellent stability, representing a promising concept for the future development of tailored HSLs for TPSC applications.

## Author contributions

Q. C. and G. Z. contributed equally to this work. D. Z., M. A. and Y. W. conceived the idea and designed the experiment. G. Z. synthesized materials and collected data. Q. C. conducted the solar cell fabrication and characterized perovskite films and devices. P. L., J. L., T. G. and J. L. assisted on the characterizations and fabrications. Q. C. and X. W. performed GIWAXS characterization and analysis. Q. C., G. Z. and D. Z. wrote the manuscript, and D. Z., Y. L., X. L., M. A., Y. C., C. T. and Y. W. revised the manuscript.

## Conflicts of interest

The authors declare no conflicts of interest.

## Supplementary Material

SC-017-D5SC08439C-s001

SC-017-D5SC08439C-s002

## Data Availability

The data that support the findings of this study are available from the corresponding author upon reasonable request. Supplementary information (SI) is available. See DOI: https://doi.org/10.1039/d5sc08439c.
