# Supplementary material for: A donor–acceptor zwitterion as a self-assembled hole-selective layer for highly efficient tin-based perovskite solar cells
Source: Chem Sci. 2025 Dec 29;17(8):4268–76. doi: 10.1039/d5sc08439c (PMC12805863; doi:10.1039/d5sc08439c)
Supplement: SC-017-D5SC08439C-s002 [file SC-017-D5SC08439C-s002.pdf]

Supplementary Information (SI) for Chemical Science.
This journal is © The Royal Society of Chemistry 2025

Supporting Information

Donor-Acceptor Zwitterion as Self-Assembled Hole-Selective Layer for Highly Efficient Tin-Based Perovskite Solar Cells

Qianqian Chang[a,#], Guosen Zhang[a,#], Diwei Zhang[a,], Peng Lin[a], Jingjing Li[a], Xurang Wang[a], Tianci Gu[a], Jingying Lin[a], Yuan Lin[a], Xiaozhen Li[a], Mingwei An[a,*], Yu Cao[a,*], Chengbo Tian[b,*], Yang Wang[a,*]*

[a] Q. Chang, G. Zhang, D. Zhang, P. Lin, J. Li, X. Wang, T. Gu, J. Lin, Y. Lin, X. Li, M. An, Y. Cao, Y. Wang.

Strait Institute of Flexible Electronics (SIFE, Future Technologies), Fujian Key Laboratory of Flexible Electronics, College of Physics and Energy, Fujian Normal University and Strait Laboratory of Flexible Electronics (SLoFE), Fuzhou 350117, Fujian, P. R. China

E-mail: ifedwzhang@fjnu.edu.cn; ifemwan@fjnu.edu.cn; ifeyucao@fjnu.edu.cn; ifewangy@fjnu.edu.cn

[b] C. Tian

Xiamen Key Laboratory of Optoelectronic Materials and Advanced Manufacturing, Institute of Luminescent Materials and Information Displays, College of Materials Science and Engineering, Huaqiao University, Xiamen 361021, China

mail: cbtian@hqu.edu.cn

#： Q. Chang and G. Zhang contributed equally to this work.

Experimental Section

Materials. All chemicals were used as received without further purification, tin(II) iodide (SnI_2, 99.99%, 3A Materials), phenethylammonium thiocyanate (PEACSN, ≥99.5%, Adamas), tin (II) fluoride (SnF_2, 99%, Sigma-Aldrich), N,N-dimethylformamide (DMF, 99.8%, Sigma-Aldrich), dimethyl sulfoxide (DMSO, ≥99.9%, Sigma-Aldrich) and chlorobenzene (CB, 99.8%, Sigma-Aldrich). Formamidine iodide (FAI, 99.9%), methylammonium bromide (MABr, 99.9%), C61-butyric acid methyl ester (PCBM) and indium tin oxide (ITO, 7-9 Ω sq^{-1}) were purchased from Advanced Election Technology Co., Ltd. Bathocuproine (BCP, 99%) was obtained from Xi'an Yuri Solar Co., Ltd. Silver (Ag) with high purity (99.99%) was sourced from Zhongnuo Advanced Material (BeiJing) Technology Co., Ltd.

Synthesis. All reagents and chemicals are commercially purchased and are used without further purification unless otherwise stated. Solvents were purified by standard methods and dried if necessary.

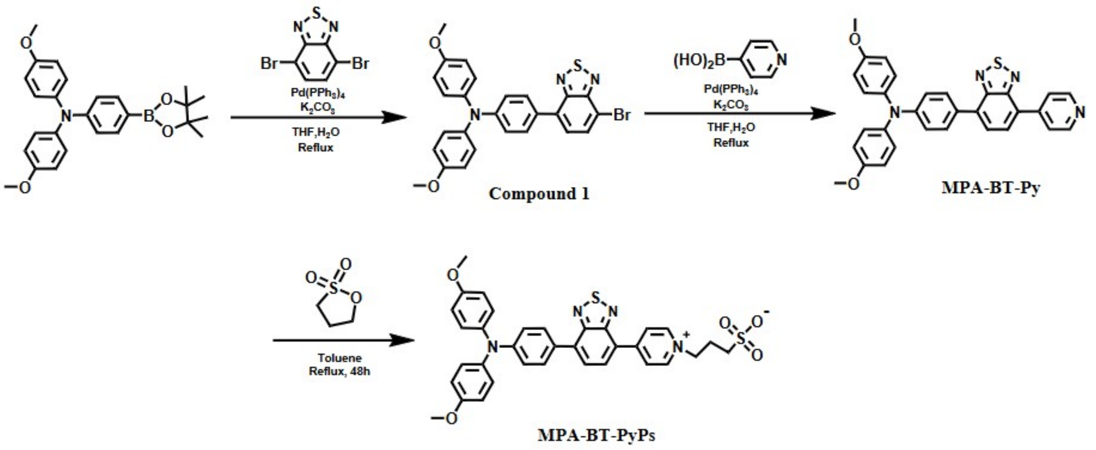

Scheme S1. Synthesis of MPA-BT-Py (Py) and MPA-BT-Py (PyPs).

Synthesis of compound 1. A mixture of 4,7-Dibromo-2,1,3-benzothiadiazole (1.29 g, 4.4 mmol), 4-Methoxy-N-(4-methoxyphenyl)-N-(4-(4,4,5,5-tetramethyl-1,3,2-dioxaborolan-2-yl)phenyl)aniline (1.73 g, 4 mmol), $Pd(PPh_3)_4$ (138.7 mg, 0.12 mmol),and potassium carbonate (829.2 mg, 6 mmol), in THF (42 mL) and water (6 mL)was heated to reflux overnight. After cooling to room temperature, the mixture was extracted with CH_2Cl_2, and the combined organic layer was dried over anhydrous Na_2SO_4. After the solvent was evaporated, the residue was purified by column chromatography (silica gel, petroleum ether: CH_2Cl_2 = 1:1 as the eluent) to

afford a red solid in 68.5% yield (1.4 g). ^{1}H NMR (400 MHz, DMSO-d_6): δ 8.05 (d, J = 7.7 Hz, 1H), 7.81 (d, J = 8.8 Hz, 2H), 7.68 (d, J = 7.7 Hz, 1H), 7.10 (d, J = 9.0 Hz, 4H), 6.95 (d, J = 9.0 Hz, 4H), 6.86 (d, J = 8.8 Hz, 2H), 3.75 (s, 6H).

Synthesis of compound MPA-BT-Py (Py). A mixture of compound 1 (311 mg, 0.6 mmol), Pyridine-4-boronic acid (88.6 mg, 0.72 mmol), $Pd(PPh_3)_4$ (35 mg, 0.03 mmol),and potassium carbonate (116.1 mg, 0.84 mmol), in THF (7.5 mL) and water (3 mL)was heated to reflux overnight. After cooling to room temperature, the mixture was extracted with CH_2Cl_2, and the combined organic layer was dried over anhydrous Na_2SO_4. After the solvent was evaporated, the residue was purified by column chromatography (silica gel, petroleum ether:CH_2Cl_2 = 1:2 as the eluent) to afford a dark red solid in 73% yield (227 mg). ^{1}H NMR (600 MHz, DMSO-d_6): δ 8.74 (d, J = 6.3 Hz, 2H), 8.10 (d, J = 7.5 Hz, 1H), 8.05 (d, 2H), 7.92 – 7.89 (m, 3H), 7.13 (d, J = 8.9 Hz, 4H), 6.96 (d, J = 9.0 Hz, 4H), 6.89 (d, J = 8.9 Hz, 2H), 3.76 (s, 6H); ^{13}C NMR (101 MHz, DMSO-d_6): δ 156.18, 153.05, 149.96, 148.95, 139.59, 133.23, 132.04, 131.44, 130.09, 128.83, 127.25, 123.35, 118.26, 115.10, 55.29. HRMS: $C_{31}H_{24}N_4O_2S$ calcd: 516.1620 found: 517.1686[M+H].

Synthesis of compound MPA-BT-PyPs (PyPs). A compound of MPA-BT-Py (232 mg, 0.45mmol), was dissolved in dried toluene (0.04 M). After the mixture was stirred for 20 min at room temperature, 1,3-propanesultone (54.6 mg, 0.45mmol) was added. Then, the reaction mixture was heated at 120 °C for 48 h under nitrogen atmosphere. After cooling to room temperature, the resultant precipitate was washed with toluene. The crude residue was collected by subsequently washed with toluene (6 mL x 1) and diethyl ether (6 mL x 3) and dried under vacuum to afford MPA-BT-PyPs (270 mg, 94.4%) as purple solid. ^{1}H NMR (600 MHz, DMSO-d_6): δ 9.15 (d, J = 7.1 Hz, 2H), 8.84 (d, J = 7.0 Hz, 2H), 8.43 (d, J = 7.6 Hz, 1H), 7.98 (d, J = 7.6 Hz, 1H), 7.94 (d, J = 8.4 Hz, 2H), 7.11 (d, J = 8.9 Hz, 4H), 6.94 (d, J = 8.9 Hz, 4H), 6.85 (d, J = 8.9 Hz, 2H), 4.74 (t, J = 7.0 Hz, 2H), 3.73 (s, 6H), 3.35 – 3.29 (m, 2H), 2.31 – 2.20 (m, 2H); ^{13}C NMR (101 MHz, DMSO-d_6) δ 156.38, 153.20, 152.71, 151.51, 149.51, 144.70, 139.29, 136.55, 132.30, 130.46, 127.47, 126.65, 126.21, 126.08, 123.65, 117.82, 115.14, 64.92, 58.97, 55.31, 15.17. HRMS: $C_{34}H_{30}N_4O_5S_2^-$ calcd: 638.1663 found: 639.1726[M+H].

Theoretical Calculations. The ground-state geometry optimization was calculated using the density functional theory (DFT) method at the B3LYP/def2-SVP level of theory with the Gaussian 09 program package. Stationary points were verified by frequency analysis.[1] The optimized structures were found to be stable. The calculated molecular electronic static potential (ESP) results were obtained with the Multiwfn 3.7 program.[2]

Characterization and Measurements. The ^1H NMR and ^{13}C NMR spectra were performed using a AVANCE NEO 400 instrument. Thermogravimetric (TGA) measurements were carried out on a METTLER TOLEDO (TGA 1 STARe System) apparatus at a heat ramp of 10 ^oC min^{-1}. The electrochemical cyclic voltammetry (CV) was measured in a 0.01 mol/L tetrabutylammonium hexafluorophosphate (Bu_4NPF_6) CH_2Cl_2 solution as the supporting electrolyte with a scan speed at 0.025 V/s. Ultraviolet-visible (UV-Vis) absorption spectra were recorded on a UH5700 spectrophotometer. Fourier transform infrared (FTIR) spectra were measured with a Thermo Fisher Nicolet IS50. The crystal structure of perovskite films were characterized by X-ray diffraction (XRD, UltimaIV). The surface composition of the perovskite film was characterized by Xray photoelectron spectroscopy (XPS, Shimadu, AXIS SUPRA+). Atomic force microscopy (AFM) and Kelvin probe force microscopy (KPFM) images were characterized by atomic force microscopy (Bruker, Icon). The contact angle images were measured with an OCA 15EC. The energy dispersive X-ray spectroscopy (EDS) images were characterized by field emission scanning electron microscopy (Hitachi, SU8010). Ultraviolet photoelectron spectroscopy (UPS) spectra were collected using ESCALAB 250Xi. Steady-state photoluminescence (PL) (excitation wavelength 460 nm) and time-resolved photoluminescence (TRPL) were conducted with Edinburgh Instruments LTD (FLS 980). The microstructure of perovskite films were observed using high-resolution field emission scanning electron microscopy (FE-SEM, SU8600). Grazing-Incidence Wide-Angle X-ray Scattering (GIWAXS) Measurements. GIWAXS measurements were carried out using a Xeuss 3.0 X-ray scattering station (Xenocs, France). The samples were illuminated with 8.04-keV radiation (λ = 1.542 Å) at an incident angle (α) of 0.2°. The sample-to-detector distance was set to 90 mm. The detector calibration was conducted using a standard reference material, and the beam

center was determined to be at (x = 248, y = 1066) pixels. The pixel size of the detector was 0.075 mm. The J-V curves were obtained at a scan rate of 0.02 V/s using a Keithley 2400 source meter under standard one sun illumination (AM 1.5G spectrum, 100 mW/cm^{-2}) within a N_2-filled glovebox. The EQE was measured by using a solar cell quantum efficiency measurement system (Model QEX10). Electrochemical impedance (EIS) and Mott-Schottky measurements were tested in the dark using an electrochemical workstation (Zennium Zahner, Germany). Space-charge limited current (SCLC) measurement was performed by a Keithley 2450 source meter. In the dark state, the J-V curves (used to evaluate conductivity) of the ITO/ HSL /Ag devices were measured using the Kestrel 2450 source meter, with the measurement range of (-1) to (+2) V. In the dark, the Agilent B1500A semiconductor characterization system is used to measure transient photovoltaic (TPV) and transient photocurrent (TPC). The dark J-V characteristics were recorded with a Keithley 2400. Long-term stability assessment was conducted on the unencapsulated Sn-based perovskite cells in an N_2 atmosphere, which were achieved through multiple repeated J-V characteristics.

Device Fabrication. The Sn-based perovskite cells were fabricated with a configuration of ITO/HSL/Perovskite/PCBM/BCP/Ag. The ITO-coated glass substrates were initially submerged in deionized water containing detergent, followed by a thorough ultrasonic cleansing in a sequence of deionized water, acetone and isopropanol for a duration of 30 minutes each. Then, the cleaned ITO glass substrates were treated with UV-O_3 for 15 minutes. The 60 μL solution of HSL (1 mg/mL in DMF) was deposited on the ITO substrate in a nitrogen atmosphere at 6000 rpm for 40 s and then annealed at 80 °C for 10minutes. The perovskite precursor solution was prepared by dissolving 128.98 mg FAI, 27.99 mg MABr, 372.52 mg SnI_2, 5mg Sn powder, 27.04 mg PEASCN and 15.67 mg SnF_2 in a mixed solvent of 800 μL DMF and 200 μL DMSO and then stirring for about 10 h. Before spin-coating, the tin perovskite precursor was filtered through a 0.22 μm polytetrafluoroethylene (PTFE) filter. The 60 μL tin perovskite precursor solution was spin-coated on the ITO/HSLs substrate at 8000 rpm for 60 s, at 15s after which chlorobenzene as antisolvent was injected and then annealed at 80 °C for 10 minutes. Then, PCBM solution (18mg/mL in CB) was spin-coated onto the tin

perovskite film at 2000 rpm for 30 seconds and then annealed at 80 °C for 10 minutes. After cooling down to room temperature, deposited BCP (7 nm) and Ag (100 nm) on the perovskite film to form the solar cell fabrication in a vacuum chamber. The effective cell area was 0.075 cm^2.

Supplementary Note 1. PS energy level calculation

The work function was calculated using the equation:

$$\Phi = 21.22 \text{ eV} - E_{\text{cut-off}}. \qquad (1)$$

The valance band maximum (VBM) was calculated using the equation[3,4]:

$$E_{\text{VBM}} = 21.22 \text{ eV} - E_{\text{cut-off}} + E_{\text{onset}} \qquad (2)$$

The conduction band minimum (CBM) was calculated using the equation:

$$E_{\text{CBM}} = E_{\text{VBM}} + E_{\text{g}} \qquad (3)$$

where E_{g} represents the optical bandgap of the sample, which was calculated according to the onset wavelength of the absorption spectrum

Supplementary Note 2. The films state densification of molecules by varying the scan rates through CV cyclic voltammetry, according to the Randles-Sevcik equation:

$$i_p = \frac{n^2F^2}{4RTN_A}\nu A\Gamma^* \qquad (4)$$

i_p represent the peak current (A), n is the number of electrons transferred, F is the Faraday constant (96485.33 C mol^{-1}), R is the universal gas constant (8.314 J K^{-1} mol^{-1}), ν is the voltage scan rate (V s^{-1}), T is the temperature, N_A is the Avogadro constant, A is the electrode area, and Γ^* is the areal density.

Supplementary Note 3. Calculation of the defect density (N_t)

Trap densities (N_t) of devices employed on hole-only devices with the structures of ITO/ HSL/perovskite/PTAA/Ag. According to the formula:

$$N_t = \frac{2\varepsilon_0\varepsilon_r V_{TEL}}{eL^2} \qquad (5)$$

Where ε_0 is the vacuum permittivity, ε_r is the relative dielectric constant, V_{TFL} is the transition voltage between ohmic and trap-filling regions, e is the electron charge, and L is the tin perovskite film thickness.

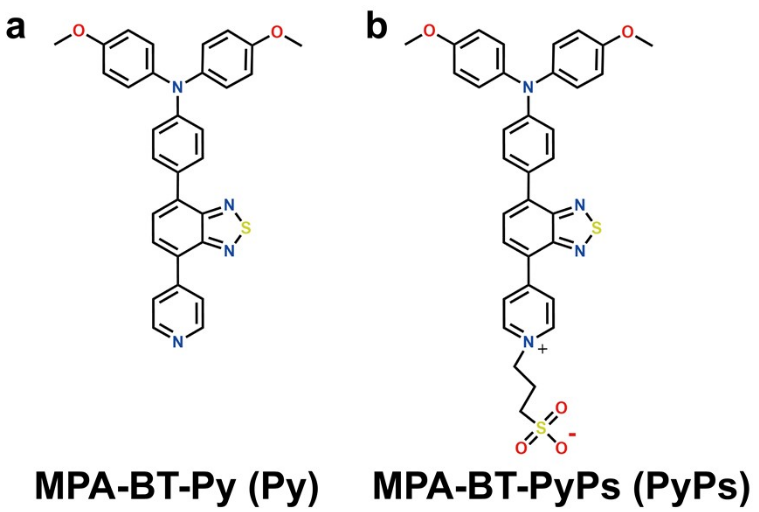

Fig. S1. The chemical structures for (a) MPA-BT-Py and (b) MPA-BT-PyPs.

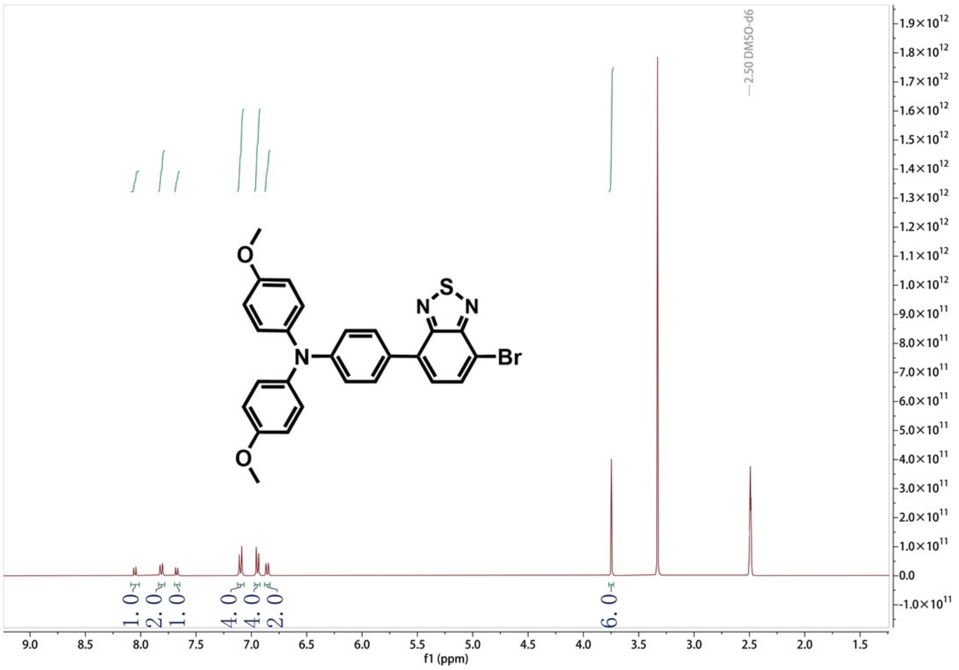

Fig. S2. ^{1}H NMR spectrum of compound 1 in DMSO-*d6*.

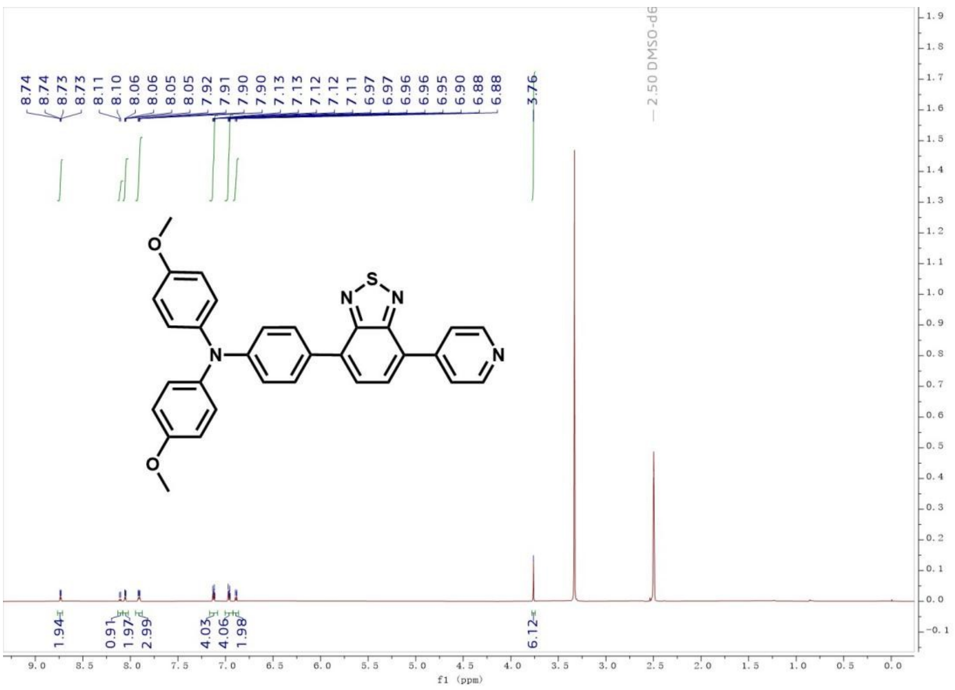

Fig. S3. ^{1}H NMR spectrum of MPA-BT-Py in DMSO-*d6*.

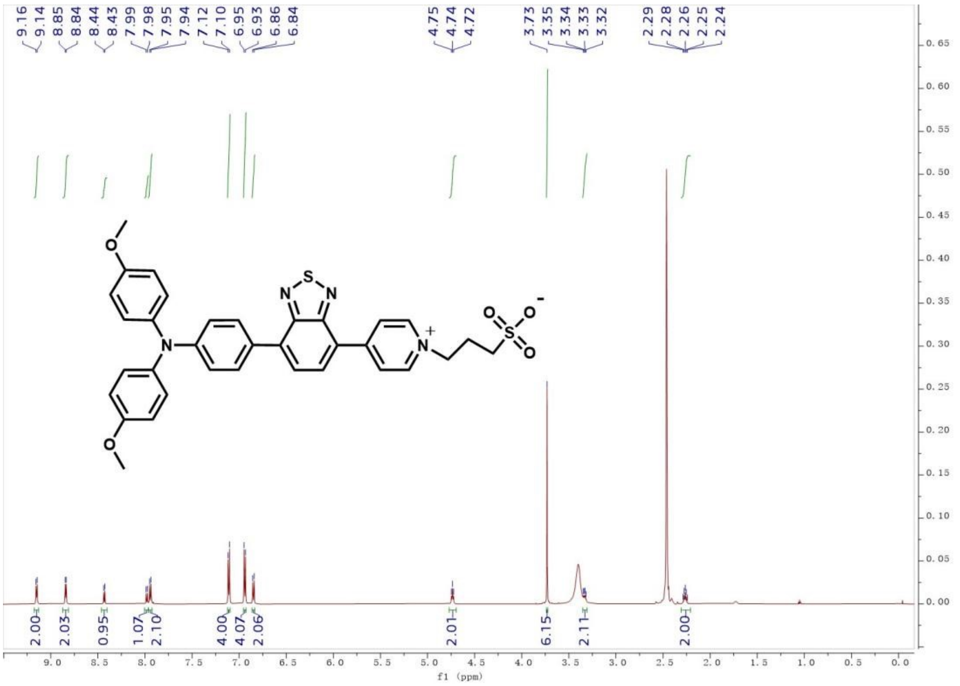

Fig. S4. ^{1}H NMR spectrum of MPA-BT-PyPs in DMSO-*d*6.

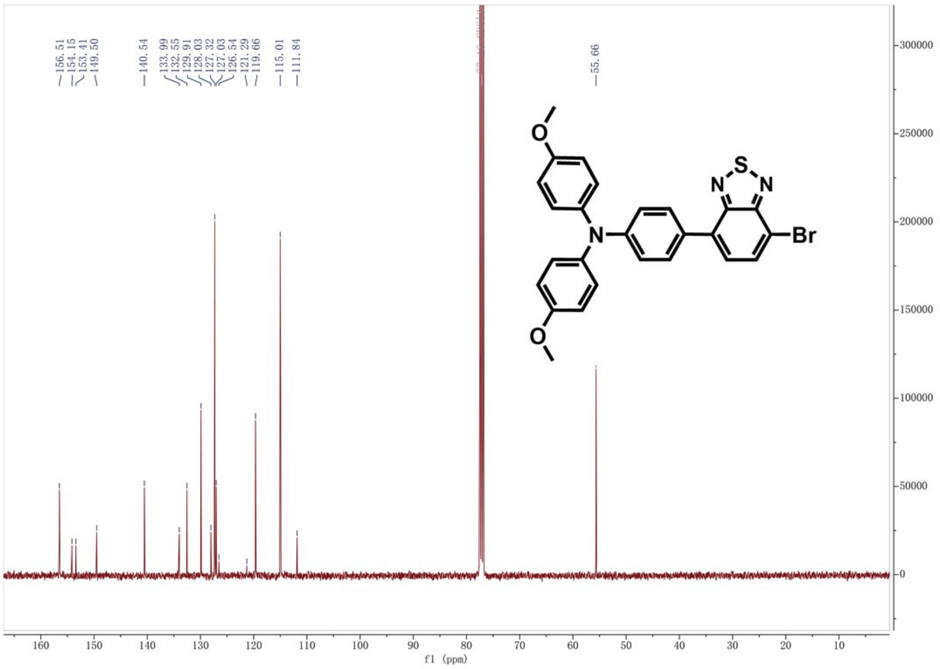

Fig. S5. ^{13}C NMR spectrum of compound 1 in $CDCl_3$-*d*.

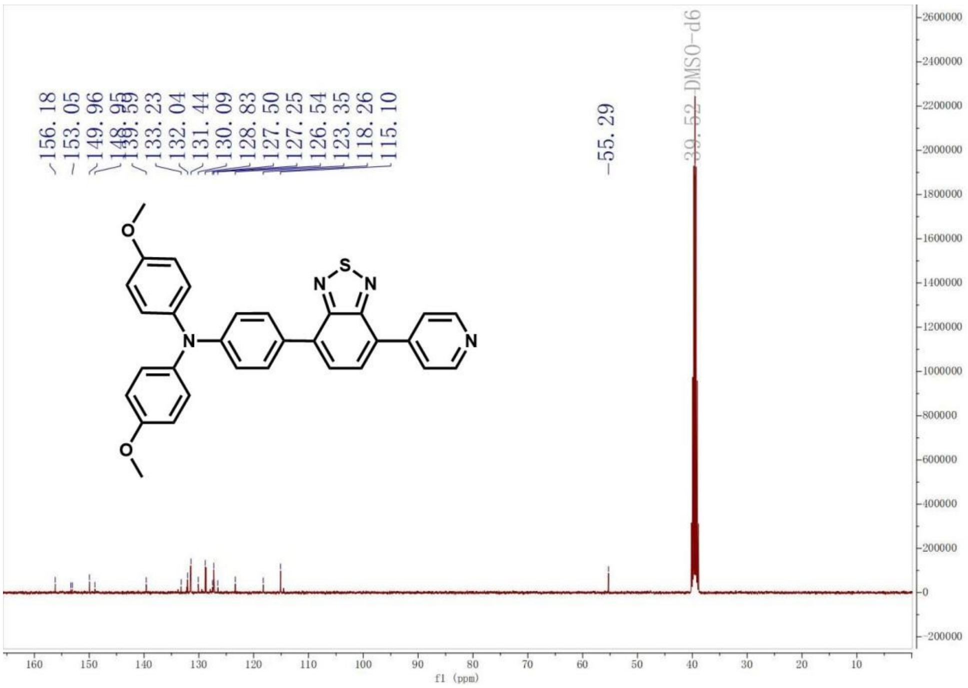

Fig. S6. ^{13}C NMR spectrum of MPA-BT-Py in DMSO-*d6*.

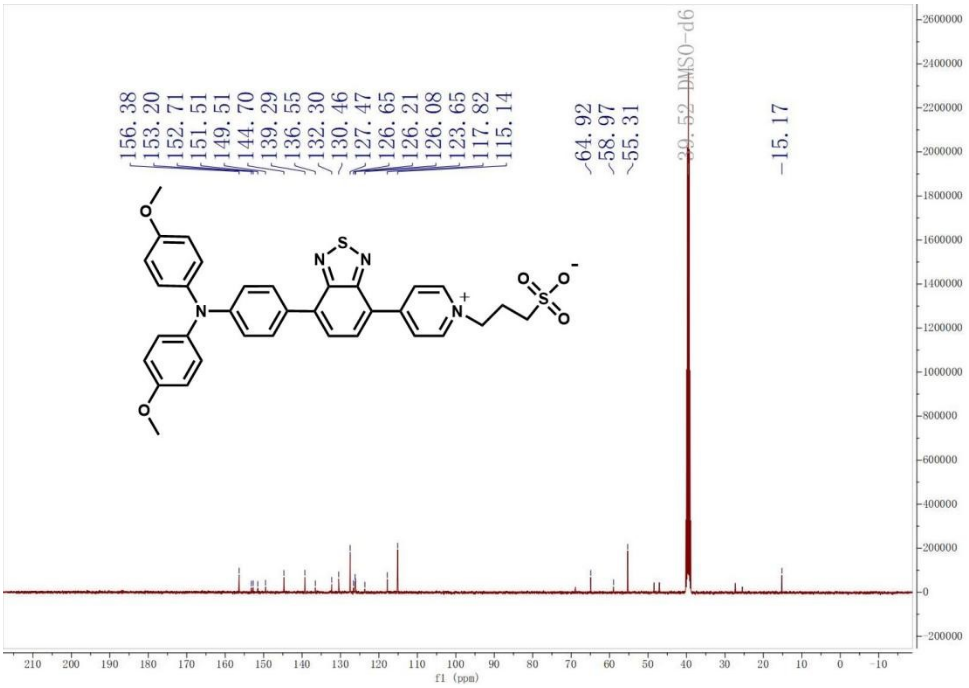

Fig. S7. ^{13}C NMR spectrum of MPA-BT-PyPs in DMSO-d_6.

MPA-BT-Py:

HRMS: $C_{31}H_{24}N_4O_2S$ calcd: 516.1620 found: 517.1686[M+H].

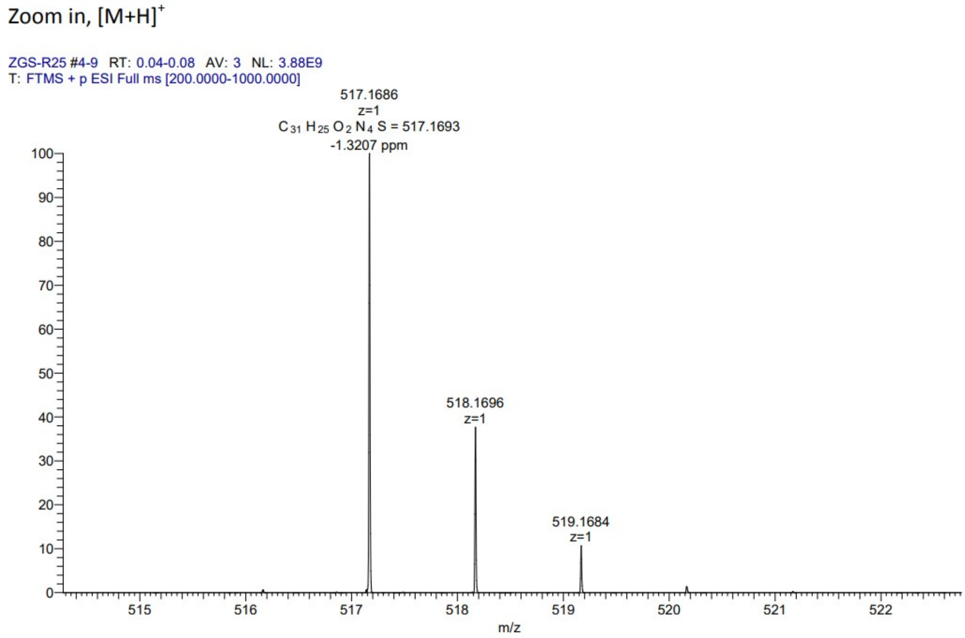

Fig. S8. HRMS spectrum of MPA-BT-Py.

MPA-BT-PyPs:

HRMS: $C_{34}H_{30}N_4O_5S_2^-$ calcd: 638.1663 found: 639.1726[M+H].

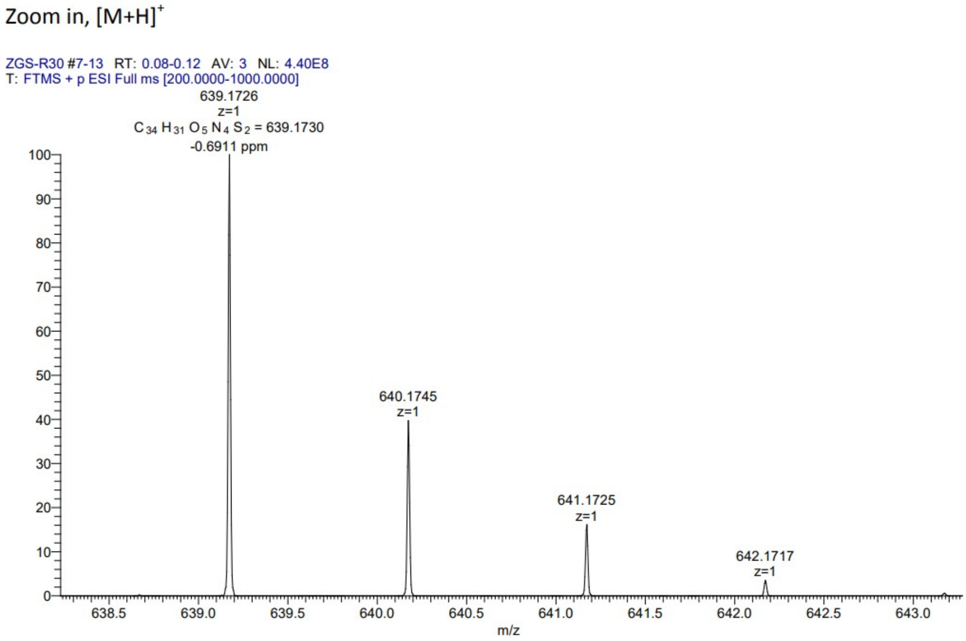

Fig. S9. HRMS spectrum of MPA-BT-PyPs.

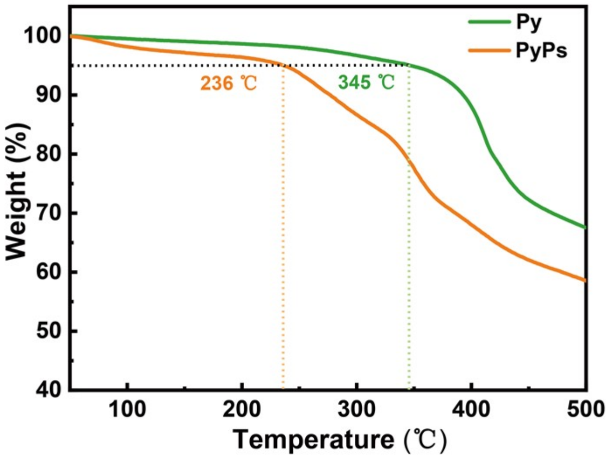

Fig. S10. The TGA curves of HSLs measured with a heating rate of 10 ^{o}C min^{-1} under a nitrogen condition.

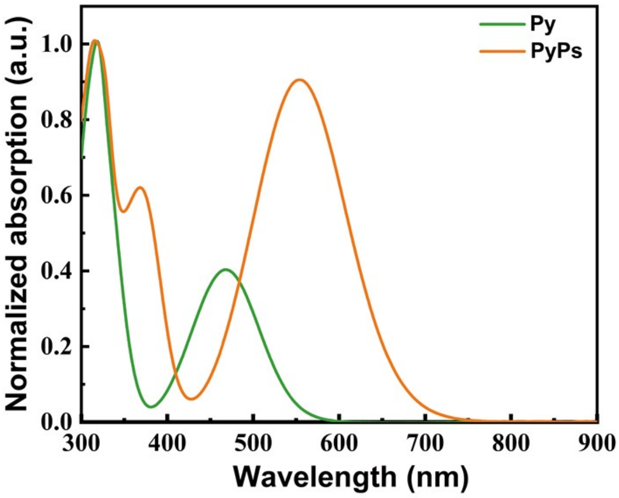

Fig. S11. UV-visible absorption spectra of the HSLs in CH_2Cl_2 solution (10^{-5}M).

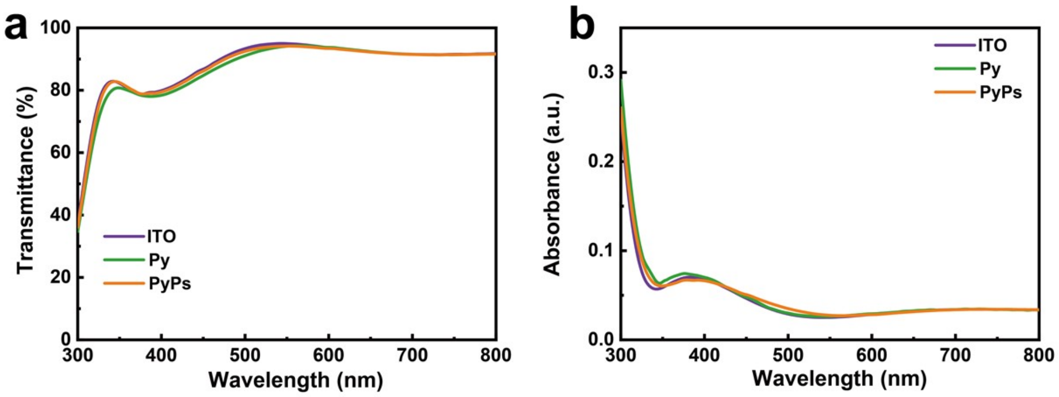

Fig. S12. (a) Transmittance and (b) absorption spectra of ITO, Py and PyPs.

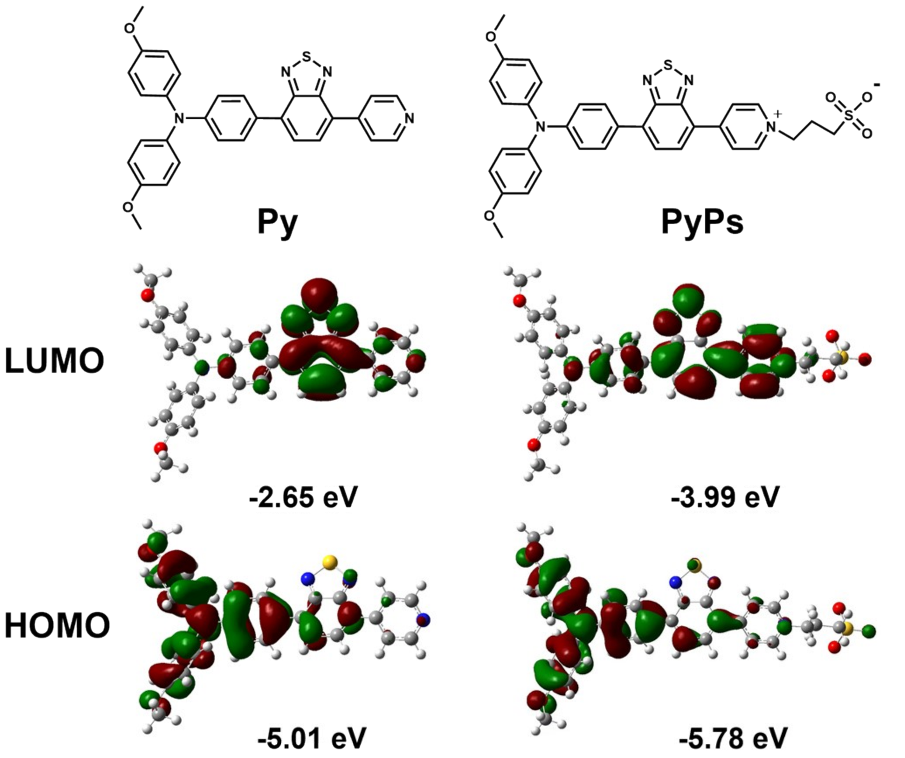

Fig. S13. Commutated frontier molecular orbital distributions of Py and PyPs by B3LYP/6-31G (d, p).

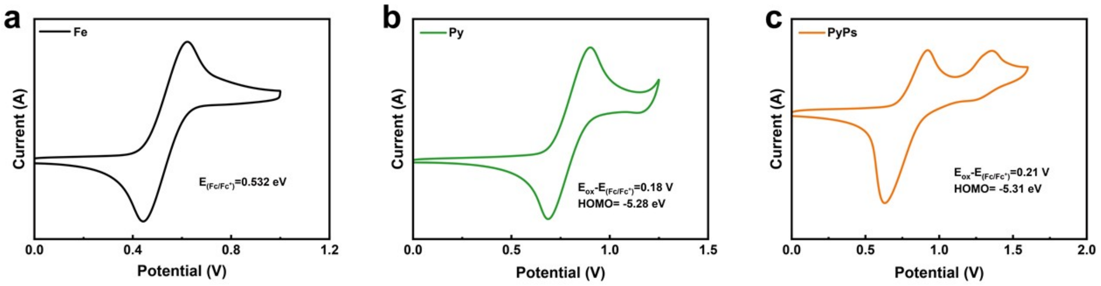

Fig. S14. Cyclic voltammetry characterized oxidation potential of phosphates corresponding to (a) reference and (b, c) Py and PyPs in (5×10^{-4} M) in CH_2Cl_2 with 0.1M Bu_4NPF_6 as electrolyte, glassy carbon and platinum wire as working and counter electrodes and Ag/AgCl as reference electrode; scanning rate was 100 mV s^{-1}; Ferrocene was used as external reference and the potentials were presented by reference to $E_{1/2}(Fc/Fc^+)$.

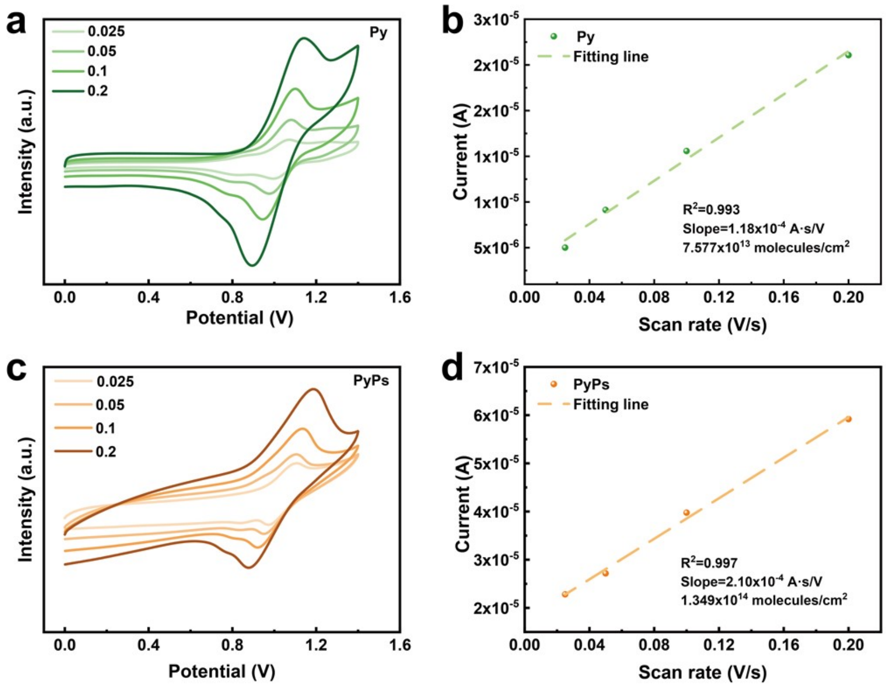

Fig. S15. Cyclic voltammograms of Py film (a) and PyPs film (c) as the working electrode measured in Ar-saturated o-DCB solution under different voltage scan rates. The relationship between the oxidative peak current and the voltage scan rate for (b, d) ITO/ HSLs. The dashed line represents a linear fit to the raw data.

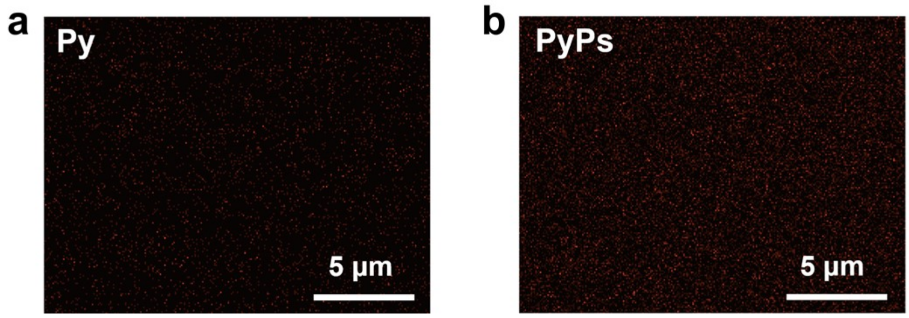

Fig. S16. EDS mapping of S element distribution of HSLs on ITO substrates.

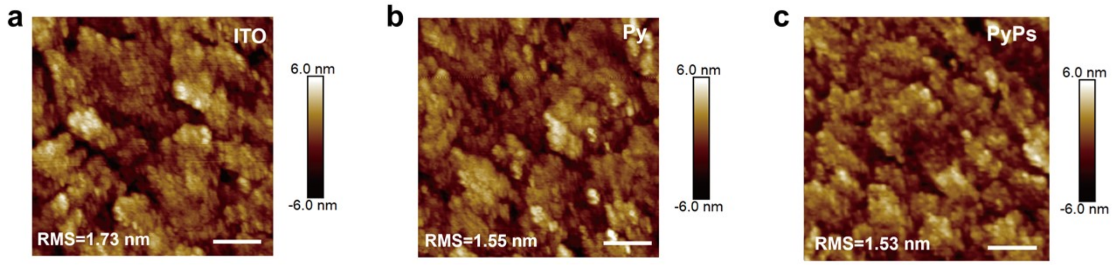

Fig. S17. AFM images of (a) ITO, (b) Py and (c) PyPs films (scale bar 500 nm).

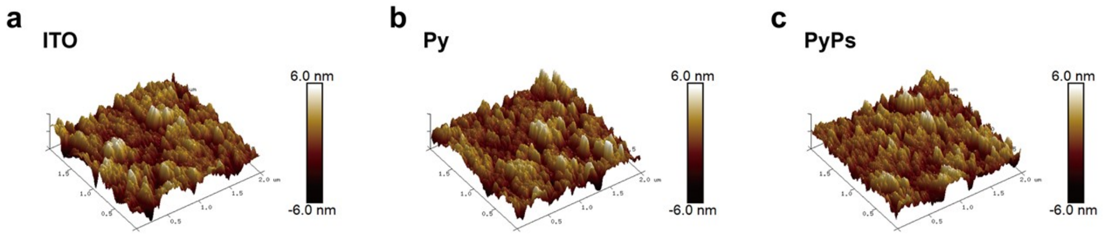

Fig. S18. AFM 3D images of (a) ITO, (b) Py and (c) PyPs films.

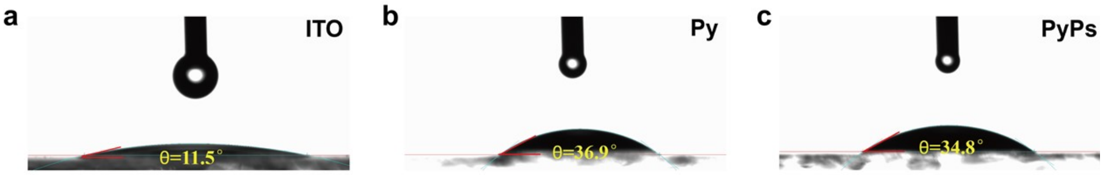

Fig. S19. Contact angles of H_2O on (a) ITO, (b) Py and (c) PyPs substrates.

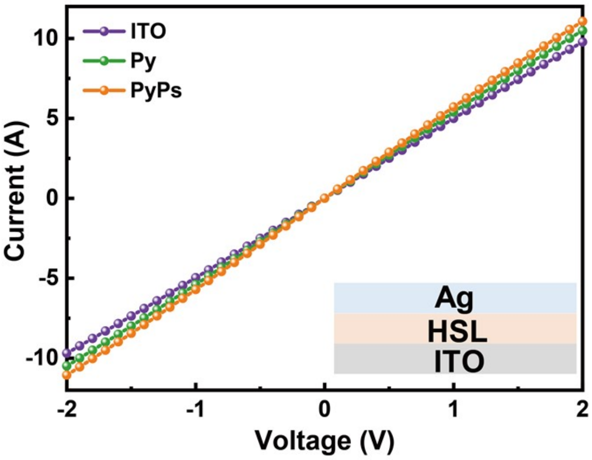

Fig. S20. The conductivity of ITO without and with HSLs modification.

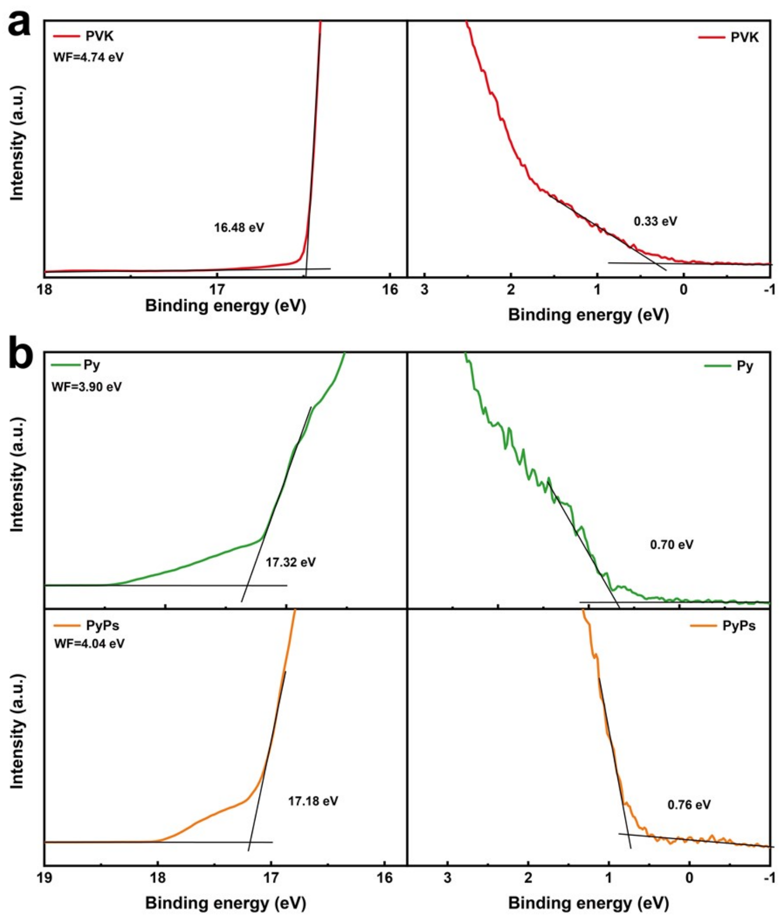

Fig. S21. The UPS spectra of (a) perovskite films and (b) HSLs films.

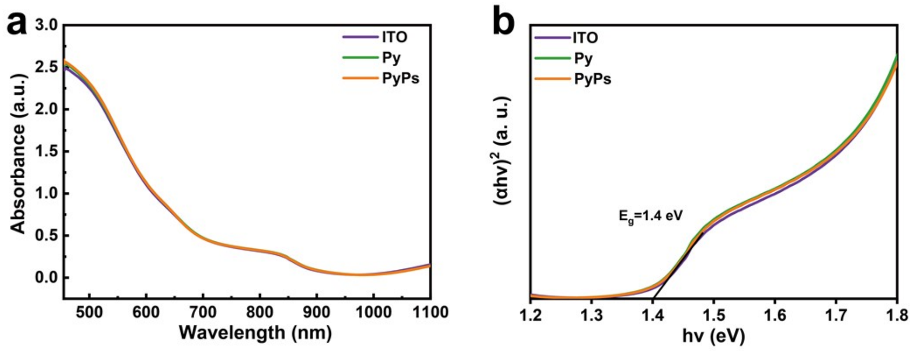

Fig. S22. (a) Absorption spectra and (b) Tauc plots Value of perovskite on ITO, Py and PyPs films.

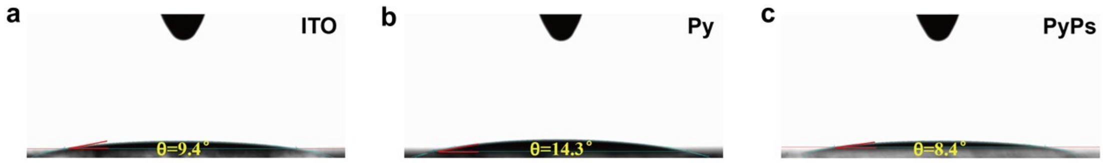

Fig. S23. Contact angles of perovskite precursor solution on (a) ITO, (b) Py and (c) PyPs substrates.

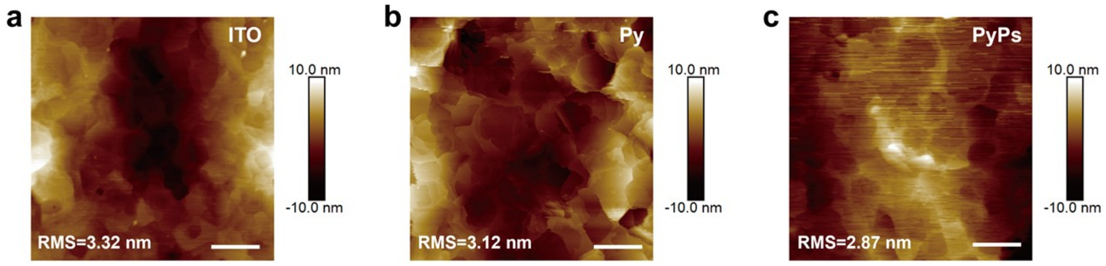

Fig. S24. AFM images for (a) ITO, (b) Py and (c) PyPs perovskite films.

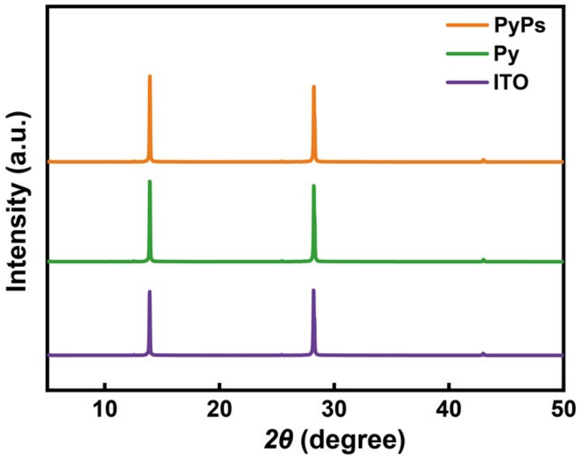

Fig. S25. XRD patterns of tin perovskite films on ITO, Py and PyPs.

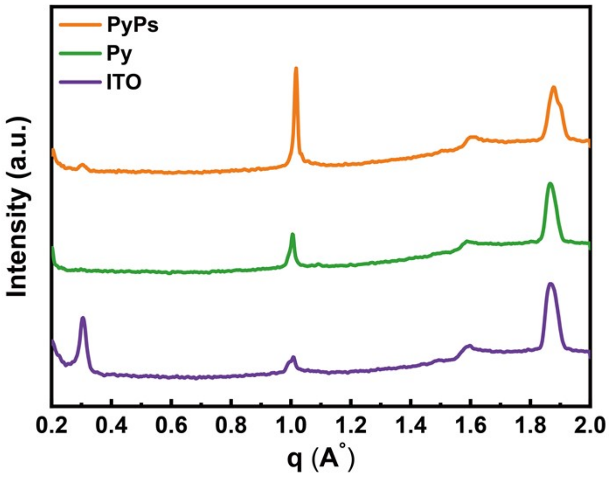

Fig. S26. 1D line profiles extracted from GIWAXS spectra for ITO, Py, and PyPs-based perovskite films.

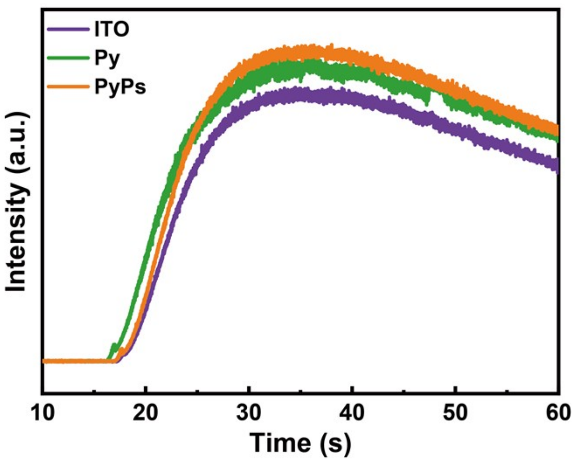

Fig. S27. The contour plot of PL spectra at the wavelength of 885 nm.

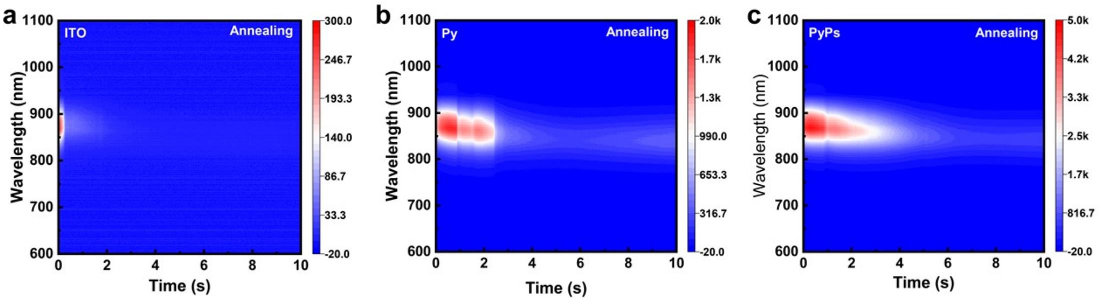

Fig. S28. The annealing process of the (a) ITO, (b) Py and (c) PyPs based perovskite films.

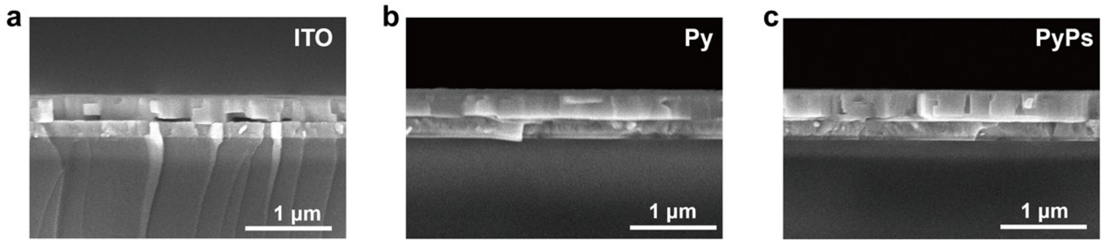

Fig. S29. Cross-sectional SEM images of the corresponding perovskite films on (a) ITO, (b) Py and (c) PyPs substrates.

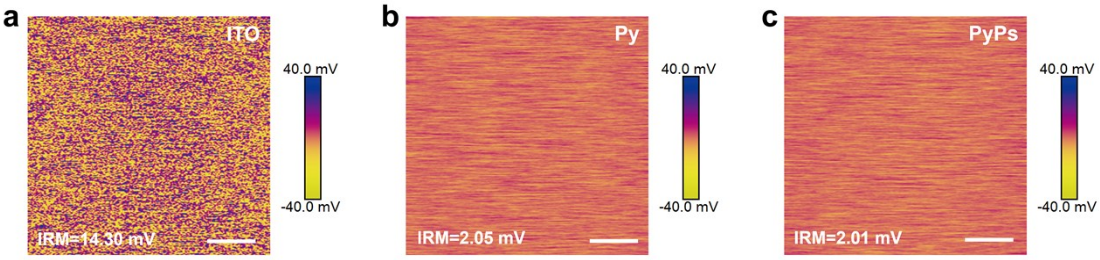

Fig. S30. KPFM images for (a) ITO, (b) Py and (c) PyPs based perovskite films.

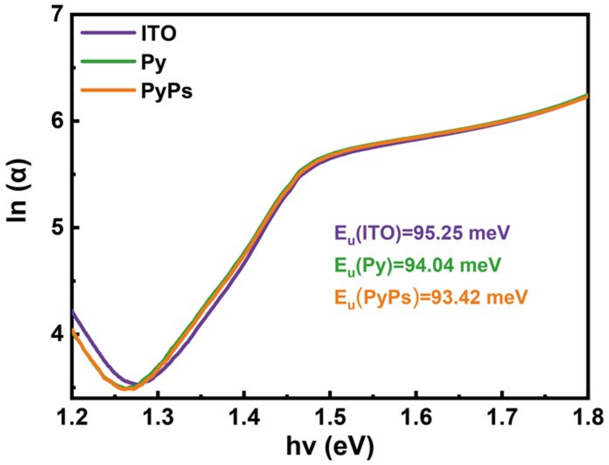

Fig. S31. The Urbach energy (E_U) Value of perovskite films for ITO, Py and PyPs.

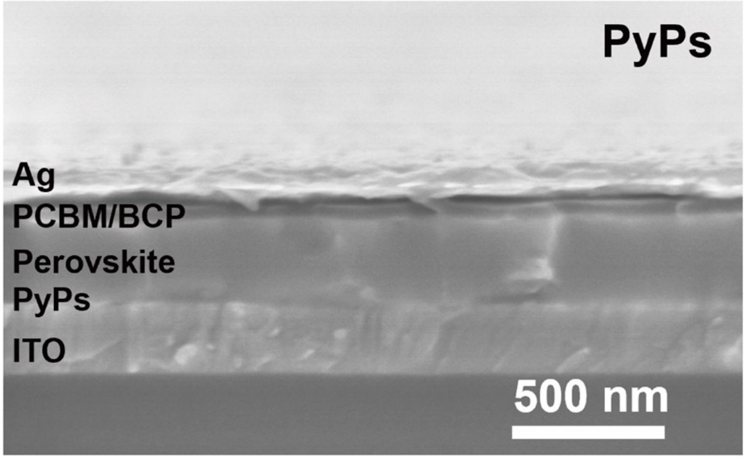

Fig. S32. Cross-sectional SEM images of the corresponding perovskite device.

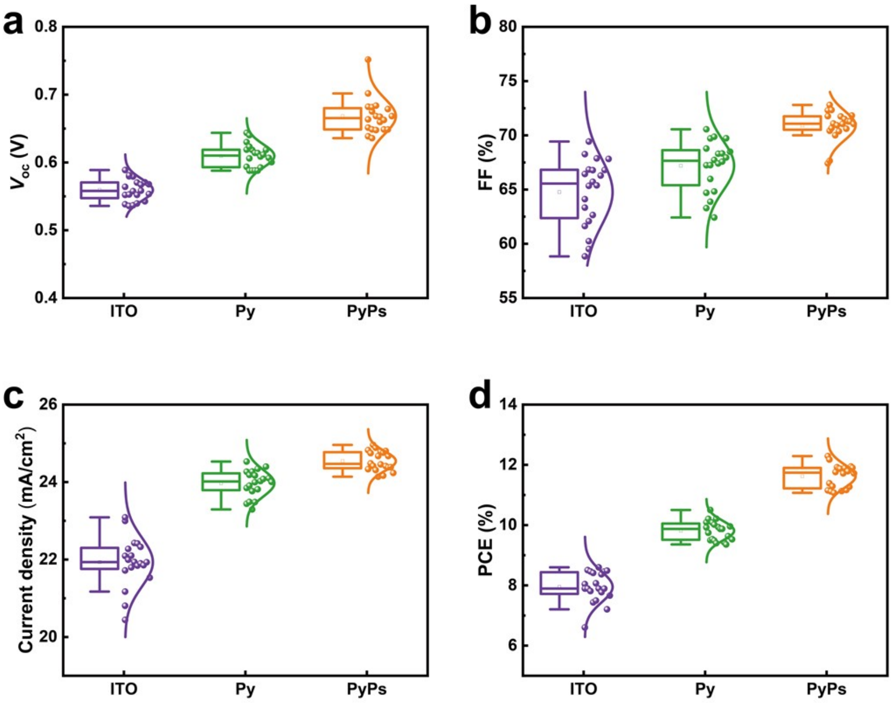

Fig. S33. Box charts of (a) V_{OC}, (b) FF, (c) J_{SC}, and (d) PCE of the TPSCs based on ITO, Py and PyPs films (20 individual devices).

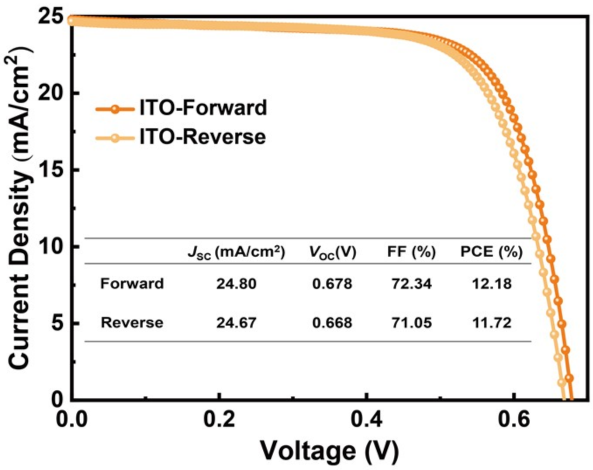

Fig. S34. Effect of hysteresis of the PyPs based device.

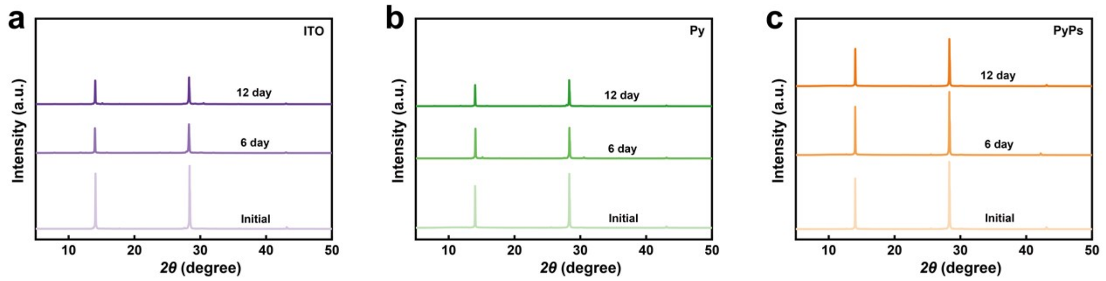

Fig. S35. XRD patterns of fresh and aged perovskite films on (a) ITO, (b) Py and (c) PyPs with in N_2 at 30 ± 5 °C for 12 days.

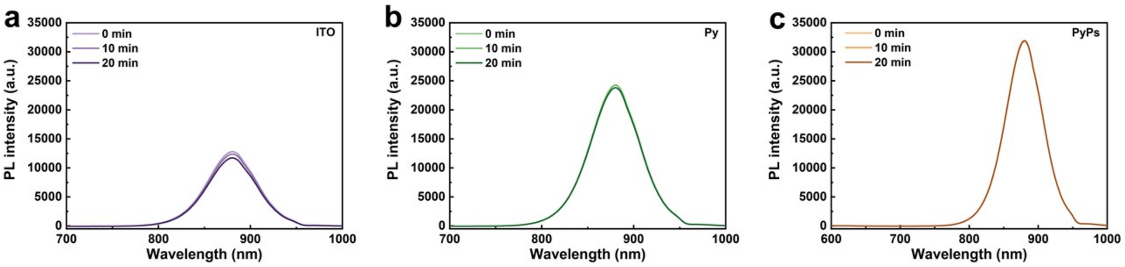

Fig. S36. PL spectra of perovskite films on (a) ITO, (b) Py and (c) PyPs under continuous laser irradiation conditions (marked as one sun based on the equivalent J_{SC} of the device).

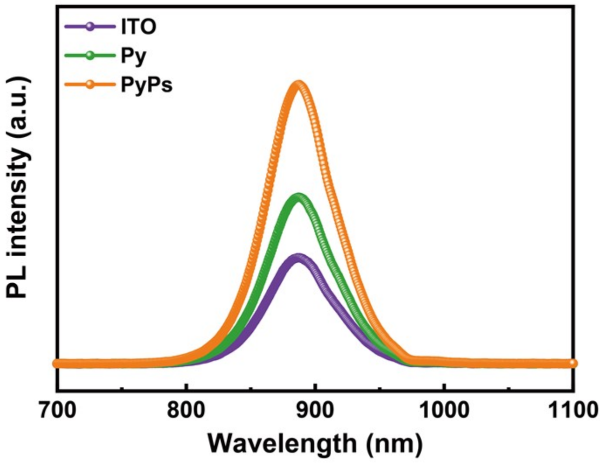

Fig. S37. PL spectrum of perovskite deposited on ITO, Py and PyPs upon excitation from perovskite side.

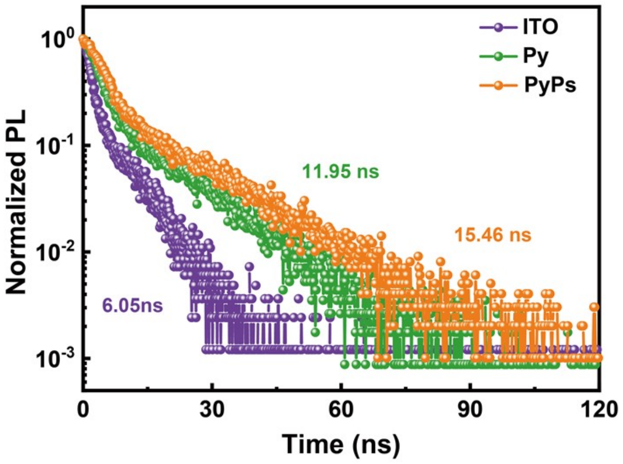

Fig. S38. TRPL spectrum of perovskite deposited on ITO, Py and PyPs upon excitation from perovskite side.

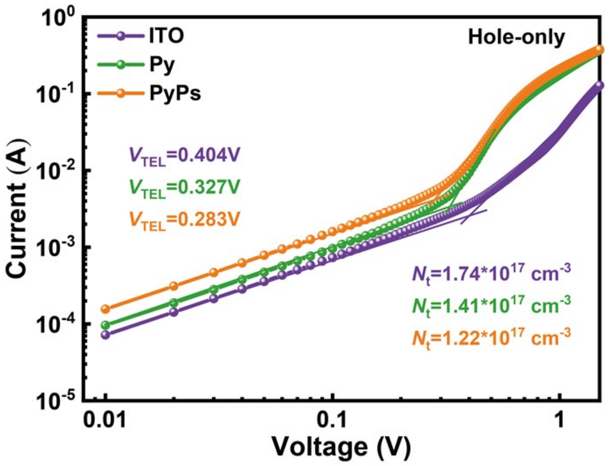

Fig. S39. SCLC curves for hole-only devices.

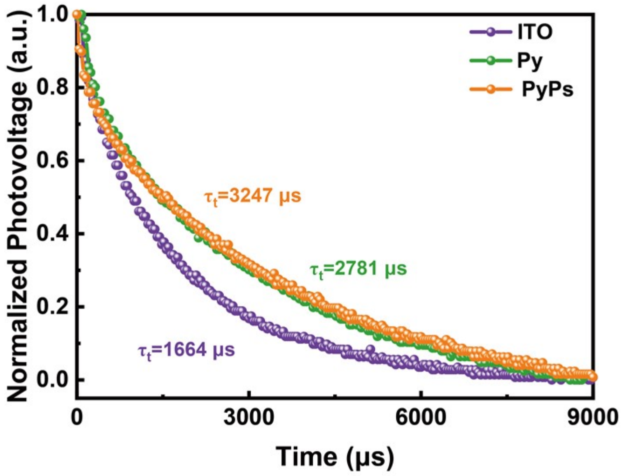

Fig. S40. Normalized transient photovoltage (TPV) spectra.

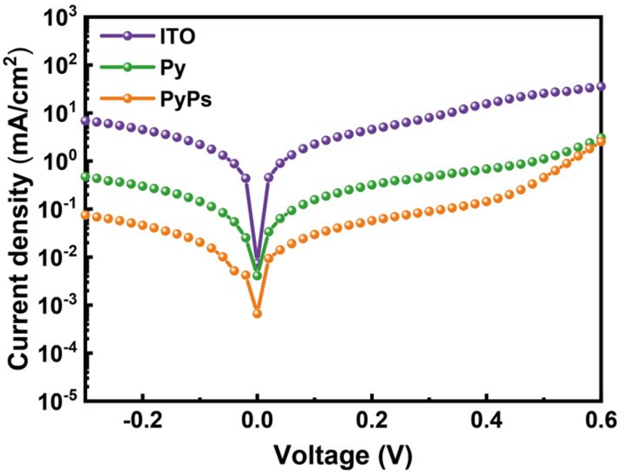

Fig. S41. Dark *J-V* curves.

Table S1. Summary of energy level parameters for Py, PyPs and PVK films.

	E_{CBM} (eV)	E_{VBM} (eV)	E_g (eV)	Work Function (eV)
Py	-2.17	-4.60	2.43	3.90
PyPs	-2.49	-4.80	2.31	4.04
PVK	-3.67	-5.07	1.40	4.74

Table S2. The fitting results of XPS spectra of perovskite films.

Name		Sn $3d_{5/2}$		Sn $3d_{3/2}$		Sn^{2+} (%)	Sn^{4+} (%)
		Sn^{2+}	Sn^{4+}	Sn^{2+}	Sn^{4+}		
ITO	Position (V)	486.3	494.7	486.8	495.2	57.39	42.61
	Peak area (V)	48756.18	34495.45	29419.48	23553.11		
Py	Position (V)	486.3	494.7	486.9	495.2	66.34	33.66
	Peak area (V)	54277.50	27575.82	39163.89	19841.47		
PyPs	Position (V)	486.5	494.8	487.3	495.3	75.03	24.97
	Peak area (V)	65427.12	21705.82	41644.68	13934.44		

Table S3. Photovoltaic parameters of the ITO devices.

Device	J_{SC} (mA/cm^2)	V_{OC} (V)	FF (%)	PCE (%)
1	23.00	0.552	63.34	8.04
2	22.09	0.564	68.27	8.51
3	22.27	0.553	64.12	7.89
4	22.10	0.580	61.64	7.90
5	21.72	0.579	62.09	7.81
6	22.00	0.589	65.34	8.46
7	21.80	0.581	66.44	8.41
8	20.81	0.570	62.65	7.44
9	21.94	0.567	60.24	7.50
10	22.43	0.538	66.84	8.07
11	22.42	0.536	65.72	7.89
12	23.09	0.558	66.79	8.60
13	22.33	0.5528	67.87	8.37
14	21.84	0.537	66.29	7.77
15	21.90	0.539	66.81	7.89
16	21.85	0.571	67.82	8.46
17	21.93	0.558	58.84	7.21
18	20.44	0.543	59.52	6.60
19	21.53	0.568	69.43	8.49
20	21.17	0.553	65.39	7.66

Table S4. Photovoltaic parameters of the Py devices.

Device	J_{SC} (mA/cm^2)	V_{OC} (V)	FF (%)	PCE (%)
1	23.85	0.619	67.24	9.92
2	23.91	0.630	64.70	9.75
3	23.77	0.644	65.98	10.09
4	23.44	0.640	63.29	9.50
5	24.27	0.606	64.82	9.53
6	24.00	0.618	68.78	10.21
7	24.19	0.614	63.88	9.49
8	24.53	0.614	62.42	9.40
9	24.27	0.609	67.25	9.94
10	24.17	0.623	69.72	10.50
11	24.34	0.612	67.73	10.08
12	24.03	0.619	67.40	10.02
13	24.08	0.607	67.58	9.87
14	23.81	0.594	69.87	9.88
15	23.49	0.588	68.30	9.43
16	23.29	0.588	68.32	9.36
17	24.40	0.600	69.73	10.21
18	24.10	0.588	67.96	9.63
19	24.00	0.588	70.56	9.96
20	23.48	0.593	68.49	9.53

Table S5. Photovoltaic parameters of the PyPs devices.

Device	J_{SC} (mA/cm^2)	V_{OC} (V)	FF (%)	PCE (%)
1	24.33	0.639	71.77	11.15
2	24.82	0.651	70.427	11.39
3	24.75	0.636	70.67	11.12
4	24.48	0.649	71.09	11.29
5	24.42	0.648	70.01	11.07
6	24.31	0.682	70.93	11.76
7	24.14	0.702	70.38	11.92
8	24.96	0.664	71.74	11.88
9	24.89	0.660	72.30	11.87
10	24.46	0.675	67.41	11.13
11	24.17	0.660	70.65	11.27
12	24.67	0.668	71.05	11.72
13	24.41	0.682	71.50	11.90
14	24.38	0.649	70.61	11.17
15	24.78	0.667	71.29	11.79
16	24.38	0.649	71.28	11.27
17	24.76	0.663	72.80	11.95
18	24.24	0.684	71.83	11.90
19	24.80	0.679	72.34	12.18
20	24.67	0.668	71.05	11.72

Table S6. Summary of key properties of HSLs TPSCs, including the photovoltaic parameters presented in Figure 4c.

Year	HSLs				Perovskite			Device				
	Name	Substrate WF (eV)	E_{HOMO}/E_{VBM} (eV)	CA (deg)	Deposition	E_g (eV)	E_{VBM} (eV)	Structure	V_{OC} (V)	J_{SC} (mA/cm^2)	FF (%)	PCE (%)
2021[5]	MeO-2PACz	4.6	-5.1	55[a]	2-step method	1.41[c]	-4.9	ITO/HSL/perovskite/C60/BCP/Ag	0.475	20.3	67.3	6.5
2022[6]	PTAA	5.48	-5.1	5.97[b]	2-step method	1.4	-5.2	ITO/HSL/perovskite/C60/BCP/Ag	-	-	-	8.3
2023[7]	TQxD	4.115	-5.09	19.4[b]	2-step method	1.4	-5.2	ITO/HSL/perovskite/C60/BCP/Ag	0.574	21.05	68.8	8.3
2023[8]	PPr-SBT-14	3.96	-5.07	11.13[b]	2-step method	1.4	-5.2	ITO/HSL/perovskite/C60/BCP/Ag	0.51	22.37	66.1	7.6
2023[9]	MeO-2PACz	-	-5.3	47.07[a]	1-step method	1.4	-4.9	ITO/HSL/perovskite/C60/BCP/Ag	0.638	14.3	64	5.8
2023[10]	AB	4.8	-4.8	6.52[b]	2-step method	1.43[d]		ITO/HSL/perovskite/C60/BCP/Ag	0.534	19.92	71.9	7.6
2024[11]	TPAT-CA	3.72	-5.12	11.86[b]	2-step method	1.4	-5.2	ITO/HSL/perovskite/C60/BCP/Ag	0.58	19.2	72.6	8.1
2025[12]	PTzBr	5.08	-5.08	6.1[b]	2-step method	1.4	-5.12	ITO/HSL/perovskite/C60/BCP/Ag	0.548	19.19	74.7	7.8
2025[13]	MC-4	3.6	-5.19	15.57[b]	2-step method	1.4	-5.2	ITO/HSL/perovskite/C60/BCP/Ag	0.631	19.83	69.15	8.65
2025[14]	Th-2EPT	4.78	-	69[a]	1-step method	-	-	ITO/HSL/perovskite/C60/BCP/Ag	0.63	18.8	-	8.2
This work	PyPs	4.04	-4.8	8.4[b]	1-step method	1.4[c]	-5.07	ITO/HSL/perovskite/PCBM/BCP/Ag	0.678	24.8	72.34	12.18

[a]Measured by DI water. [b]Measured by the perovskite precursor. [c]Approximated by the absorption spectra.[d] Approximated by the photoluminescence spectra.

Table S7. Summary of the PL lifetimes from fitting curves of the TRPL decay curves.

Name	τ_1 (ns)	A_1 (%)	τ_2 (ns)	A_2 (%)	τ_{avg} (ns)
ITO	2.58	63.2	15.09	36.8	12.82
Py	1.66	80.8	7.75	19.2	6.37
PyPs	0.43	63.4	2.44	36.6	1.98

The TRPL decay spectra were fitted by a biexponential function:

$$y = y_0 + A_1 e^{-x/\tau_1} + A_2 e^{-x/\tau_2}$$

and the average decay lifetime was calculated with function:

$$\tau_{avg} = \frac{A_1\tau_1^2 + A_2\tau_2^2}{A_1\tau_1 + A_2\tau_2}$$

Table S8. Summary of the PL lifetimes from fitting curves of the TRPL decay curves.

Name	τ_1 (ns)	A_1 (%)	τ_2 (ns)	A_2 (%)	τ_{avg} (ns)
ITO	1.86	87.4	10.97	12.6	6.05
Py	3.83	88.0	22.18	12.0	11.95
PyPs	5.16	86.9	27.36	13.1	15.46

References

[1] J. V. M. J. Frisch, G. W. Trucks, H. B. Schlegel, G. E. Scuseria, M. A. Robb, J. R. Cheeseman, G. Scalmani, V. Barone, G. A. Petersson, H. Nakatsuji, X. Li, M. Caricato, A. Marenich, J. Bloino, B. G. Janesko, R. Gomperts, B. Mennucci, H. P. Hratchian, J. V. Car, Gaussian 09, Revision A.1; Gaussian, Inc.: Wallingford, CT, 2009.

[2] T. Lu, F. Chen, *J. Comput. Chem.* 2012, **33**, 580-592.

[3] Y. Wang, W. Chen, L. Wang, B. Tu, T. Chen, B. Liu, K. Yang, C. W. Koh, X. Zhang, H. Sun, G. Chen, X. Feng, H. Y. Woo, A. B. Djurišić, Z. He, X. Guo, *Adv. Mater.* 2019, **31**, 1902781.

[4] M. Zhao, W. M. Gu, K. J. Jiang, X. Jiao, K. Gong, F. Li, X. Zhou, Y. Song, *Angew. Chem. Int. Ed.* 2024, **64**, e202418176.

[5] D. Song, S. Narra, M.-Y. Li, J.-S. Lin, E. W.-G. Diau, *ACS Energy Lett.* 2021, **6**, 4179-4186.

[6] C.-H. Kuan, G.-S. Luo, S. Narra, S. Maity, H. Hiramatsu, Y.-W. Tsai, J.-M. Lin, C.-H. Hou, J.-J. Shyue, E. Wei-Guang Diau, *Chem. Eng. J.* 2022, **450**, 138037.

[7] S. N. Afraj, C. H. Kuan, J. S. Lin, J. S. Ni, A. Velusamy, M. C. Chen, E. W. G. Diau, *Adv. Funct. Mater.* 2023, **33**, 2213939.

[8] C. H. Kuan, R. Balasaravanan, S. M. Hsu, J. S. Ni, Y. T. Tsai, Z. X. Zhang, M. C. Chen, E. W. G. Diau, *Adv. Mater.* 2023, **35**, 2300681.

[9] E. Aktas, I. Poli, C. Ponti, G. Li, A. Olivati, D. Di Girolamo, F. A. Alharthi, M. Li, E. Palomares, A. Petrozza, A. Abate, *ACS Energy Lett.* 2023, **8**, 5170-5174.

[10] A. Abid, P. Rajamanickam, E. Wei-Guang Diau, *Chem. Eng. J.* 2023, **477**, 146755.

[11] S. N. Afraj, C. H. Kuan, H. L. Cheng, Y. X. Wang, C. L. Liu, Y. S. Shih, J. M. Lin, Y. W. Tsai, M. C. Chen, E. W. G. Diau, *Small* 2024, **21**, 2408638.

[12] A. Abid, A. Velusamy, S. N. Afraj, W. Pervez, T.-Y. Su, S.-H. Hong, C.-L. Liu, M.-C. Chen, E. W.-G. Diau,. *J. Mater. Chem. A* 2025, **13**, 9252-9264.

[13] C. H. Kuan, C. L. Mai, V. Saravanan, T. C. Lin, Y. S. Shih, C. H. Kuo, M. C. Tsai, C. Y. Yeh, E. W. G. Diau, *Small* 2025, **21**, 2504259.

[14] V. Stacchini, M. Rastgoo, M. Marčinskas, C. Frasca, K. Morita, L. Frohloff, A. Treglia, T. W. Gries, O. Karalis, V. Getautis, F. Ruske, A. Petrozza, N. Koch, H. Hempel, T. Malinauskas, A. Abate, A. Musiienko, *Adv. Energy Mater.* 2025, **15**, 2500841.
